# A Computational Systems Analyses to Identify Biomarkers and Mechanistic Link in Psoriasis and Cutaneous Squamous Cell Carcinoma

**DOI:** 10.3389/fimmu.2021.662528

**Published:** 2021-06-18

**Authors:** Sidra Adil, Rehan Zafar Paracha, Salma Tariq, Maryum Nisar, Sadaf Ijaz, Amnah Siddiqa, Zamir Hussain, Afreenish Amir

**Affiliations:** ^1^ Research Center for Modeling and Simulation, National University of Sciences and Technology, Islamabad, Pakistan; ^2^ Jackson Laboratory for Genomic Medicine, Farmington, CT, United States; ^3^ National Institute of Health (Pakistan), Islamabad, Pakistan

**Keywords:** microarray data analysis, RNA-seq data analysis, psoriasis, cutaneous squamous cell carcinoma, pathway analysis, biomarkers, therapeutic target, differentially expressed genes

## Abstract

Psoriasis is the most common and chronic skin disease that affects individuals from every age group. The rate of psoriasis is increasing over the time in both developed and developing countries. Studies have revealed the possibility of association of psoriasis with skin cancers, particularly non-melanoma skin cancers (NMSC), which, include basal cell carcinoma and cutaneous squamous cell carcinoma (cSCC). There is a need to analyze the disease at molecular level to propose potential biomarkers and therapeutic targets in comparison to cSCC. Therefore, the second analyzed disease of this study is cSCC. It is the second most common prevalent skin cancer all over the world with the potential to metastasize and recur. There is an urge to validate the proposed biomarkers and discover new potential biomarkers as well. In order to achieve the goals and objectives of the study, microarray and RNA-sequencing data analyses were performed followed by network analysis. Afterwards, quantitative systems biology was implemented to analyze the results at a holistic level. The aim was to predict the molecular patterns that can lead psoriasis to cancer. The current study proposed potential biomarkers and therapeutic targets for psoriasis and cSCC. IL-17 signaling pathway is also identified as significant pathway in both diseases. Moreover, the current study proposed that autoimmune pathology, neutrophil recruitment, and immunity to extracellular pathogens are sensitive towards MAPKs (MAPK13 and MAPK14) and genes for AP-1 (FOSL1 and FOS). Therefore, these genes should be further studied in gene knock down based studies as they may play significant role in leading psoriasis towards cancer.

## Introduction

Psoriasis is one of the most common and chronic skin diseases (1). It is characterized by rough scales on the skin. It could appear on any part of the body and it affects both children and adults (1). About 2-3% individuals are affected by psoriasis worldwide, that means more than 125 million people suffer from psoriasis (2). Males and females are equally targeted by psoriasis i.e., male to female ratio is 1:1. The usual onset of the disease is reported to be between 18 and 39 years, or between 50 and 69 years (2). The onset age of the disease is associated with genetic and epigenetic factors (2). The incidence rate of psoriasis is also observed to increase with time (3). The pathogenesis of psoriasis is still unclear and there are two hypotheses regarding it (4). The first hypothesis assumes that in psoriasis the immune system of the body appears to be hyperactive to an extent that it attacks itself (4); which is the characteristic of the autoimmune disease. Therefore, psoriasis shows characteristics of an autoimmune disorder (4, 5). According to the second hypothesis, the life cycle of the skin cells speeds up in psoriasis from 28 days to 3-6 days, which results in excessive cell growth (4, 6). It eventually causes the formation of dead skin cells. Despite of being shed off, these dead cells accumulate at the surface of the skin and form lesions and patches (6). Frequently, red patches or dry scales appear on elbows, knees, and scalps (7). In an individual’s lifetime psoriasis often occur repeatedly (7). The exact cause and cure of psoriasis has not yet been discovered. However, multiple factors including environmental & genetic factors and immune system of the body are considered for it (8). It is predicted that environmental factors like cold and dry weather could trigger the symptoms of psoriasis (9). Moreover, the symptoms of psoriasis could appear after certain infections like strep throat and even in stress (9). Both genetic and epigenetic factors play the substantial role in the formation and exacerbation of the symptoms of the disease (10). Genetic risk factors are HLA-Cw6, mutations in CARD14 (10). CARD14 is caspase recruitment domain family member 14 gene. Obesity, stress, smoking, alcohol consumption, HIV infection, and certain medications serve as the epigenetic factors of the disease (11).

Association of psoriasis with other comorbidities, like cardiovascular diseases, diabetes, and metabolic syndrome, has been observed (12). An interesting study (13) was conducted for analyzing the implication of cutaneous squamous cell carcinoma (cSCC) antigen with psoriasis. This study was based on the fact that serum (fluid after blood coagulation) examination of psoriasis patients always revealed the presence of cSCC antigen. The study confirmed the concentration and over-expression of cSCC antigen in psoriatic lesions. A transcriptome level analysis was performed to reveal common patterns in cSCC and psoriasis (14). In this experiment, expression levels in cSCC were compared with multiple cutaneous disorders by performing microarray analysis. The comparison of cSCC with psoriasis (psoriasis vulgaris) revealed some genes depicting same expression behavior by up regulation in both disease states of cSCC and psoriasis. Subsequently, RT (real time) reverse transcription PCR (polymerase chain reaction) along with immunohistochemistry were also performed to validate the differential expression of genes attained after comparison in both disease states of cSCC and psoriasis. The common over-expressed genes found in both cSCC and psoriasis were: DEFB4 (defensin B4), SERPINB3, STAT1, K16 (keratin 16), WNT5A, and CEACAM5. In another study (15), histopathological examination of a biopsy of a psoriasis patient revealed the development of cSCC. That patient showed negative family history regarding psoriasis and only received homeopathic treatment for psoriasis.

Studies have revealed the possibility of association of psoriasis with skin cancers, particularly non melanoma skin cancer (NMSC), which include basal cell carcinoma (BCC) and squamous cell carcinoma (SCC) (12, 16). A cohort study (1988-2011) (16) was conducted for estimating malignancy rates in psoriasis patients treated with systemic therapy. This study reported that there exists a higher rate of developing cSCC among psoriasis patients who were treated with biologics as a treatment. The speculation regarding the association of psoriasis with NMSC was built on the fact that immunosuppressors and ultraviolet phototherapy are used for treating psoriasis (12, 16). Moreover, the long-lasting inflammatory nature of psoriasis might also escalate the threat of NMSC in psoriasis patients (12). That is why, the second studied disease in this study is cSCC.

Most commonly, skin cancer is characterized by the abnormal growths of the skin (17). A sore that does not heal or takes time to heal could also be the indicator of skin cancer. Symptoms of skin cancer vary differently for its different subtypes. For instance, it could appear as small, smooth lumps. However, in some cases skin cancer appears as scaly, hard lumps that often bleed (17). Skin cancer is divided into melanoma and non-melanoma categories. Basal Cell Carcinoma (BCC) and Squamous Cell Carcinoma (SCC) set up non-melanoma skin cancer (17, 18). Squamous Cell Carcinoma is also known as cutaneous squamous cell carcinoma (cSCC). cSCC is the second most prevalent non-melanoma skin cancer (19, 20) and has the potential to recur/revert (19). It is mostly reported to appear in the Caucasian population (21). Furthermore, cSCC also accounts for about 20% of all the cutaneous cancers (19, 20, 22, 23). Regardless of the low occurrence of cutaneous SCC, it is responsible for the majority of the metastasis in disease, eventually causing deaths (24). It is stated to be counted in the list of topmost expensive cancers in the US (United States) (24). The prevalence rate of the cSCC is also vastly growing annually (25). Cutaneous SCC is caused by malignant multiplication and spread of epidermal keratinocytes (26). Often it appears on the sun exposed areas of the body; like face, head, hands, dorsal side of forearms and neck regions (17), and is more prevalent in elderly males (26). It has also been observed and reported that the particular rough patches resulted from sun exposure (aka Actinic Keratosis) also transform or lead to cSCC (17, 26, 27). Light skin tone, ultraviolet radiations, arsenic compounds, and immunosuppression are the reported risk factors and are found to increase the likelihood of developing cSCC (28). Some of the common pathological and clinical factors for the examination of the disease are age, gender, ethnicity, tumor size, histology, organ transplantation, psoriasis, leukemia, history of previously injured skin & nodal metastasis (29). Along with patient’s characteristics, tumor site & size, tumor depth, and histologic features accounts for recurrence and metastasis risks of cSCC (26). Genes which are reported to be frequently mutated in cSCC are TP53, CDKN2A, Ras, and NOTCH1 (20).

With the increasing annual incidence of psoriasis and cSCC, there is a great urge to comprehend and compare the diseases at the molecular level by interpreting the genomic data. There is a need to validate known biomarkers of psoriasis and cSCC through high throughput data. Moreover, common therapeutic target for both the diseases is not yet discovered. It has also been discussed that prolonged psoriasis could lead to cSCC (15). According to our understanding, the association of both the diseases has not been comparatively analyzed at molecular level in detail. In the current study, microarray and RNA-seq data analyses have been performed on the publicly available datasets of both psoriasis and cSCC to reveal the common and uncommon patterns in both the diseases.

## Materials and Methods

**Figure 1:**
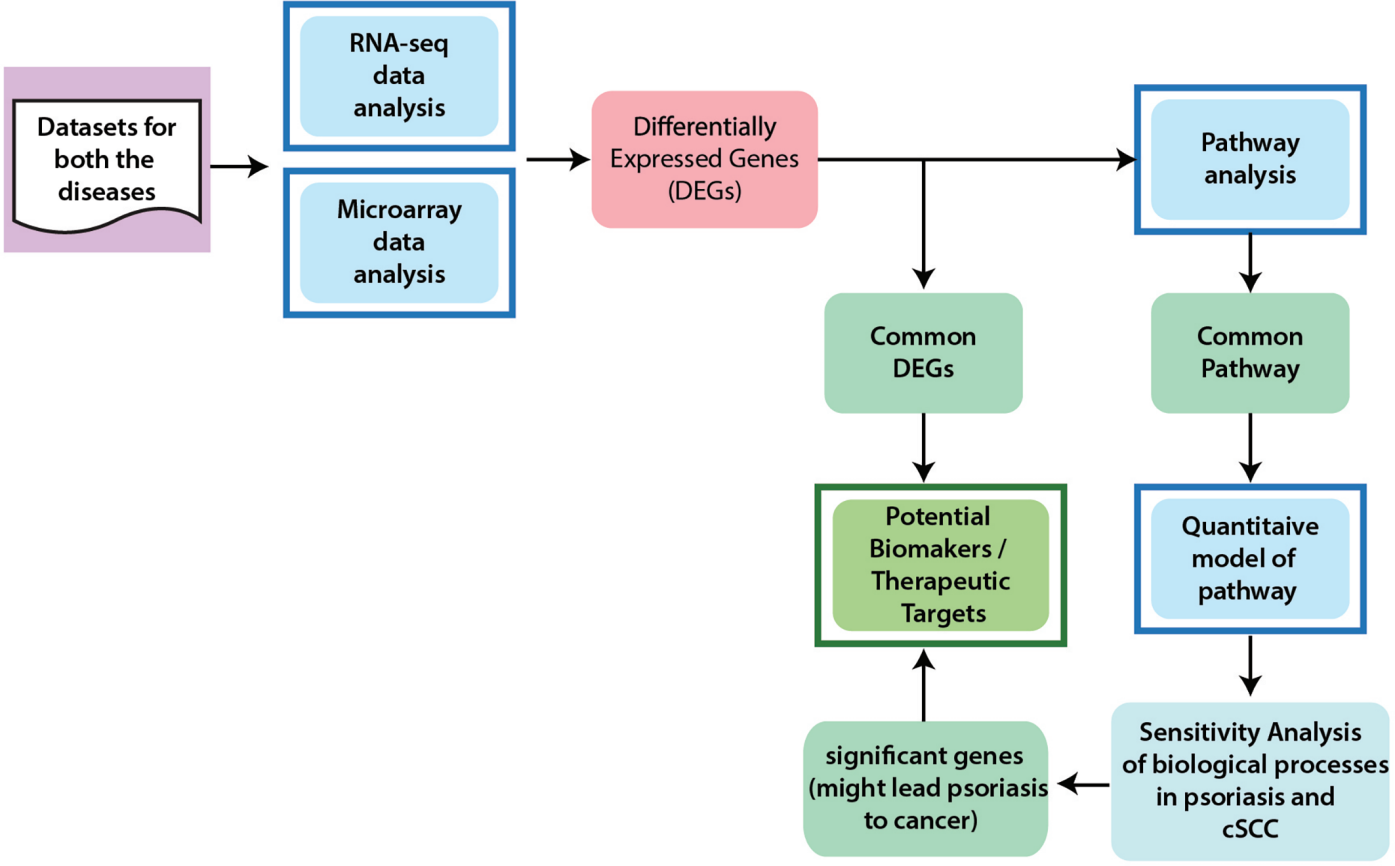
General Workflow of the Project. In order to achieve the aims and objectives of this study, integrated data analysis of microarray and RNA-seq were performed for cSCC and psoriasis. From differential expression analysis, common DEGs between psoriasis and cSCC were identified that are proposed as potential biomarkers and therapeutic targets for both the diseases. Pathway analysis was then performed to analyze the disrupted pathways and their possible relation with the disease. The significant pathways (at p-value <= 0.05) from every dataset were then compared for common pathway. As a result of this comparison, one common pathway, named IL-17 signaling pathway, was identified. Afterwards, quantitative systems biology was implemented to analyze the results at a holistic level. A sub-section of complete IL-17 pathway was modeled by using SimBiology application in Matlab (30). The average expressions values of DEGs that were present in the pathway were calculated for microarray datasets of psoriasis and cSCC. The model was then simulated by adding the average expression values. After simulation of the model, sensitivity analysis was performed to analyze the response of the biological processes to model quantities (DEGs and genes). Sensitivity analysis of the biological processes of both psoriasis and cSCC revealed significant genes that might lead psoriasis to cancer and could also be proposed as potential biomarkers and therapeutic targets of both the diseases.

The workflow to identify potential biomarkers, therapeutic targets, and to comprehend the association between cSCC and psoriasis, is given in **Figure 1** and described below in detail.

### Datasets Inclusion Criteria

Publicly available datasets of human origin, free from drugs, cell lines, and mutations, were selected from EMBL-EBI (https://www.ebi.ac.uk/) and GEO (https://www.ncbi.nlm.nih.gov/geo/) databases against query words like, psoriasis and cutaneous squamous cell carcinoma (cSCC). Additionally, to make the statistical analysis significant, those datasets with enough number of samples were selected. The **Table 1** shows the details of analyzed datasets of both the diseases.

**Table 1:** Microarray and RNA-seq datasets.

Sr. no.	Accession no.	Organism	Platform	RNA-type	Disease	Samples	Region
Microarray Datasets							
1	E-GEOD-32868	Homo Sapiens	Affymetrix	miRNA	cSCC	4	USA
2	E-GEOD-34536	Homo Sapiens	Agilent	miRNA	cSCC	14	Germany
3	E-GEOD-42677	Homo Sapiens	Affymetrix	mRNA	cSCC	20	USA
4	E-GEOD-75343	Homo Sapiens	Affymetrix	mRNA	Psoriasis	45	USA
5	E-GEOD-50790	Homo Sapiens	Affymetrix	mRNA	Psoriasis	8	USA
6	E-GEOD-41664	Homo Sapiens	Affymetrix	mRNA	Psoriasis	76	USA
7	E-GEOD-34248	Homo Sapiens	Affymetrix	mRNA	Psoriasis	28	USA
8	E-GEOD-13355	Homo Sapiens	Affymetrix	mRNA	Psoriasis	180	USA
RNA-seq Datasets							
1	E-GEOD-41745	Homo Sapiens	Illumina	mRNA	Psoriasis	6	USA
2	E-GEOD-67785	Homo Sapiens	Illumina	mRNA	Psoriasis	28	USA
3	E-MTAB-5678	Homo Sapiens	Illumina	mRNA	cSCC	9	UK
4	GSE84293	Homo Sapiens	Illumina	mRNA	cSCC	15	USA

The First eight rows show the information regarding microarray datasets of both the diseases, while, the information regarding RNA-seq datasets is given in last four rows. All the selected datasets for RNA-seq analysis have single end (SE) data, except for the dataset E-MTAB-5678, which contains paired end (PE) data.

A total of 3 datasets were analyzed for microarray data analysis of cSCC, as given in **Table 1**. Two of these datasets, with accession numbers E-GEOD-32868 and E-GEOD-34536, are of miRNA type, containing 4 and 14 samples, respectively. The third dataset, with accession number E-GEOD-42677 is of mRNA type, containing 20 samples.

For microarray data analysis of psoriasis, a total of 5 datasets were analyzed, as given in **Table 1**. All these datasets are of mRNA type, with accession numbers E-GEOD-75343, E-GEOD-50790, E-GEOD-41664, E-GEOD-34248 and E-GEOD-13355, containing 45, 8, 76, 28 and 180 samples, respectively.

Datasets for RNA-seq analysis of both the diseases are given in **Table 1**. All datasets are of mRNA type and from illumina platform. Two datasets were analyzed for cSCC, with accession numbers E-MTAB-5678 and GSE84293, containing 9 and 15 samples, respectively. Two datasets were analyzed for psoriasis, with accession numbers E-GEOD-41745 and E-GEOD-67785, containing 6 and 28 samples, respectively.

### Differential Expression Analysis

#### Microarray Data Analysis

Microarray data analysis was performed in R (version of 3.5.1). A published maEndToEnd pipeline (31) for Affymetrix platform, was used for microarray datasets of mRNA type. The quality control (QC) of the data was performed by data visualization and filtering techniques which include box plot, principal component analysis (PCA) plot, relative log expression (RLE) plot, and Heatmap clustering analysis. PCA and heatmap clustering analysis were performed and plotted to visualize the logarithmic transformations by using Bioconductor R packages (32, 33). Afterwards, the probes having low intensity along with the low variance were removed to avoid noise in data. Then to normalize gene expression intensities quantile normalization method (34, 35) was used. The differentially expressed genes (DEGs) were identified by fitting linear model in disease samples relative to control/normal (defined groups) by using the R package *limma* (36). The R package *limma* has linear model features and is specifically designed for comparative analysis between various experimental groups. Empirical bayes variance moderation method was implemented to the data by using *eBayes* function to generate the statistical significance values for DEGs (32).

The two selected microarray datasets of cSCC (E-GEOD-32868 and E-GEOD-34536) are of miRNA type. The dataset with accession number E-GEOD-32868 is of Affymetrix platform. The analysis of that dataset was performed by modifying the maendtoend pipeline (31) by omitting the ‘Annotation step’, as annotation is not required for microarray data analysis of miRNAs. The dataset with accession number E-GEOD-34536 is from Agilent platform. The analysis of that dataset was performed by AgiMicroRna package (37) in R. The *AgiMicroRna* package integrates the RMA (robust multiarray average) algorithm for preprocessing and differential expression computation of Agilent miRNA data. The RMA algorithm implements the linear model from the R package *limma* (36). After obtaining miRNAs, their target genes were extracted from miRWalk (38). miRWalk is a publicly available database of miRNAs targets, which integrates multiple prediction programs in a user-friendly interface. For extraction of target genes, p-value was set to < 0.005 and all the prediction programs were selected.

#### RNA-Sequencing Data Analysis

A published ‘new Tuxedo’ protocol (39) for RNA-seq data analysis was followed in this study. A Unix based workstation was used for this analysis. To address the issues regarding raw data reads of RNA-seq data, like adapter contamination, over-represented or inaccurately represented sequences and base correction etc., fastp (40) preprocessor was used. After preprocessing, the pre-processed reads were aligned to the genome by using Hisat2 (41) aligner. Afterwards, StringTie (42) was used to assemble complex aligned reads. Transcripts were assembled individually for each sample of the dataset being analyzed. After successful assembling, the collective merging of transcripts from all samples was also performed by using StringTie. In order to match the output from StringTie with reference file, a specialized program named gffcompare (http://ccb.jhu.edu/software/stringtie/gff.shtml) was used. The aligned and assembled reads were then subjected to quantification by using StringTie. Quantification refers to the computation of abundances of mapped assembled transcripts. Finally, *Ballgown* package (43) of R was used for computing differential expression.

#### Parameters for Identifying DEGs

In this study, a gene is identified as differentially expressed when it crossed both the defined thresholds of p-value and fold change. Stringent thresholds of p-value (p-value < 0.005) and fold change (-1 > *Log_2_FC* | *Log_2_FC* > +1) were used between groups of normal and disease phenotypes. Less stringent thresholds of p-value (p-value < 0.05) and fold change (-0.5 > *Log_2_FC* | *Log_2_FC* > +0.5) were used when the comparisons were made between subtypes of a disease, and between non-lesional and normal phenotypes. Our reason for this is that the non-lesional skin of the psoriasis patients behave normally, and the differential expression between subtypes of a disease would be smaller as compared to the healthy counterparts. The DEGs were visually analyzed by using the R package *EnhancedVolcano* (44).

### Pathway Analysis

Pathway analysis was performed to analyze the disrupted pathways and their possible relation with both diseases, based on the number of DEGs. The enriched pathways of DEGs were identified using Enrichr (45) database and KEGG based pathways were identified. The significant pathways (at p-value <= 0.05) from every dataset were then compared for common pathway. As a result of this comparison, one common pathway, named IL-17 signaling pathway, was identified (given in **Table 4**).

### Quantitative Systems Biology

IL-17 signaling pathway was selected for further analysis, as it was common in both psoriasis and cSCC datasets (The KEGG IL-17 signaling pathway - Homo sapiens is provided in **Supplementary File 1**). The average expression values of the DEGs present in the IL-17 pathway were calculated for both psoriasis and cSCC microarray datasets, as given in **Table 5**. Quantitative systems biology was implemented by using ‘SimBiology’ application in Matlab (30). A sub-section of complete IL-17 signaling pathway was modeled in Matlab for analyzing the sensitivities of autoimmune pathology (AP), neutrophil recruitment (NR), and immunity to extracellular pathogens (IEP) towards DEGs, by using block diagram editor in SimBiology. The species were used as biological entities (genes/biological processes) in the model. The activation and inhibition reactions were then added among species in accordance with the IL-17 signaling pathway. The model was then simulated by adding the average expression values to the species (genes) as initial state values. The genes that were not differentially expressed in the pathway were set to 0.5 as initial state value. The reason we have proposed that value to be at 0.5 because it resides between the values of 0 and 1, which are the minimum and maximum values that can be settled for these genes. The biological processes being analyzed in the model were autoimmune pathology, neutrophil recruitment, and immunity to extracellular pathogens. The initial state values of these biological processes were set at zero because we wanted to analyze the response of these biological processes to model quantities (DEGs and other). Once all the initial state values were set, the model was simulated separately for DEGs in psoriasis and cSCC by using ‘SimBiology’ application in Matlab (30). As a result of simulations, graphs were generated (given in **Figure 5**).

After simulating the model, sensitivity analysis (46) was performed. We wanted to analyze the assumptions about the influences of model entities and reactions on overall model response. Sensitivity of biological processes (autoimmune pathology, neutrophil recruitment, and immunity to extracellular pathogens) in a model were analyzed against genes. All the genes were taken as input genes, whereas biological processes were taken as output. The sensitivity graphs were then generated for further analysis (given in **Figures 6** and **7**).

## Results

### Differential Expression Analysis

#### Microarray Data Analysis of Psoriasis

The microarray data analysis was performed on the selected psoriasis datasets using the methodology described comprehensively in the section “Methods”. In the current study, five mRNA type microarray data sets were selected for psoriasis as given in **Table 1**. For two datasets of psoriasis (E-GEOD-13355 & E-GEOD-75343), three phenotypic subgroups were available, which were used to draw comparison between three groups including lesional & non-lesional, lesional & normal, and non-lesional & normal.

The common genes among all the subgroups based comparisons were identified. **Supplementary Figures S1–S3** and **S4–S6** depict the volcano plots displaying DEGs between the defined subgroups of datasets E-GEOD-75343 and E-GEOD-13355, respectively. The red dots in the volcano plots are the identified DEGs. **Supplementary Figures S7–S9** depict the volcano plots displaying DEGs of E-GEOD-50790, E-GEOD-41664 and E-GEOD-34248 datasets, respectively. The number of identified DEGs from all the analyzed microarray datasets of psoriasis are given in **Table 2**. The datasets of psoriasis were compared for common genes by comparing the identified DEGs from all five analyzed datasets. As a result of this comparison, one common DEG, named s100a8, was identified. S100a8 is a protein coding gene that mainly functions in inflammatory and immune responses (47).

**Table 2:** Number of identified DEGs**/**differentially expressed miRNAs of microarray datasets.

Sr. no.	Dataset ID	Disease	RNA-type	DEGs/differentially expressed miRNAs
1	E-GEOD-13355	Psoriasis	mRNA	24 DEGs
2	E-GEOD-75343	Psoriasis	mRNA	25 DEGs
3	E-GEOD-34248	Psoriasis	mRNA	1041 DEGs
4	E-GEOD-41664	Psoriasis	mRNA	1250 DEGs
5	E-GEOD-50790	Psoriasis	mRNA	1425 DEGs
6	E-GEOD-42677	cSCC	mRNA	118 DEGs
7	E-GEOD-32868	cSCC	miRNA	6 miRNAs 884 target genes
8	E-GEOD-34536	cSCC	miRNA	12 miRNAs 1327 target genes

The target genes of identified differentially expressed miRNAs (of cSCC datasets E-GEOD-32868 and E-GEOD-34536) were extracted from miRWalk (38) database.

#### RNA-Seq Data Analysis of Psoriasis

**Figure 2:**
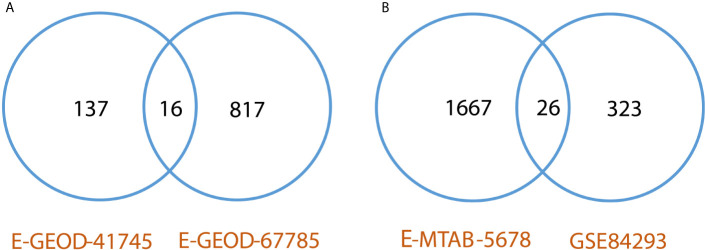
**(A)** Comparative analysis of RNA-seq datasets of psoriasis. The analysis revealed 16 common DEGs between psoriasis datasets, and, 137 and 817 unique identified DEGs from datasets E-GEOD-41745 and E-GEOD-67785, respectively. **(B)** Comparative analysis of RNA-seq datasets of cSCC. The analysis revealed 26 common DEGs between cSCC datasets, and, 1667 and 323 unique identified DEGs from datasets E-MTAB-5678 and GSE84293, respectively.

Differentially expressed genes were identified between lesional & non-lesional phenotypes in RNA-seq data analysis of psoriasis. Two datasets of psoriasis were analyzed for RNA-seq analysis as shown in **Table 1**. The number of identified DEGs from these two datasets is given in **Table 3**. From datasets E-GEOD-41745 and E-GEOD-67785, 153 and 833 DEGs were identified, respectively. **Supplementary Figures S10** and **S11** depict the volcano plots displaying DEGs between psoriatic lesional & non-lesional phenotypes of datasets E-GEOD-41745 and E-GEOD-67785, respectively. The datasets were then compared for common genes by comparing the identified DEGs from both the analyzed datasets. As a result of this comparison, 16 common DEGs were identified. The common DEGs are given in **Table 3**. This comparison also revealed that datasets E-GEOD-41745 and E-GEOD-67785 have 137 and 817 unique identified DEGs, as shown in **Figure 2**.

**Table 3:** Number of identified and symbols of common DEGs of RNA-seq datasets.

Sr. no.	Dataset ID	DEGs	Common DEGs	Common DEGs Symbols
Psoriasis Datasets				
1	E-GEOD-41745	153	16	PGM2, SOD2, SLC25A46, SERPINB4,
				DIO2, SPRR2B, IFI27, ATP12A,
2	E-GEOD-67785	833		S100A7A, LCN2, IL20RB, S100A12,
				TMPRSS11D, PI3, CD274 and IDH3A
cSCC Datasets				
1	E-MTAB-5678	1693	26	ELOVL5, GJB6, EIF1, FABP5
2	GSE84293	349		ANKRD28, NT5C, GPATCH2, EGR1
				ZFP36L2, SOX9, RUVBL1, MVD
				TNS1, S100A8, COL3A1, POSTN
				NFIB, ZFP36L1, GJB2, PRR34-AS1
				RASD1, ADH1B, RHPN2, SREBF1
				MAFF and INSIG1

#### Microarray Data Analysis of cSCC

Studies have proposed the association of psoriasis with NMSC, which include BCC and cSCC (12, 16). The chronic inflammatory nature of psoriasis (12) and use of biologics (16) might also increase the risk of cSCC in psoriasis patients. Therefore, cSCC is analyzed in the current study to interpret its association with psoriasis at the molecular level. A summary of datasets analyzed for cSCC is provided in **Table 1**. Two miRNA (E-GEOD-32868 and E-GEOD-34536) and one mRNA datasets (E-GEOD-42677) of cSCC are analyzed in this study. A total of six and 12 differentially expressed miRNAs were identified from E-GEOD-32868 and E-GEOD-34536, respectively (provided in **Supplementary Tables S12** and **S13**). The number of target genes of these differentially expressed miRNAs is given in **Table 2**. The number of identified DEGs from E-GEOD-42677 dataset is given in **Table 2**. The datasets E-GEOD-32868 and E-GEOD-34536 are composed of two phenotypes i.e., normal and cSCC. However, the dataset E-GEOD-42677 is composed of three phenotypes i.e., normal, cSCC-invasive, and cSCC-in situ. Therefore, the comparisons for finding differential expression between all the possible combinations of phenotypes within the dataset were performed for this dataset. The comparisons were between: cSCC-invasive & normal, cSCC-insitu & normal, and cSCC-invasive & cSCC-insitu groups. **Supplementary Figures S14–S16** show the volcano plots of DEGs identified in the defined comparisons of dataset E-GEOD-42677. **Supplementary Figures S12** and **S13** depict volcano plots displaying differentially expressed miRNAs between cSCC and normal phenotypes, for datasets E-GEOD-32868 and E-GEOD-34536, respectively. The datasets were compared for common genes by comparing the identified target genes of differentially expressed miRNAs with that of mRNAs. For two miRNA datasets (E-GEOD-32868 & E-GEOD-34536) 200 common DEGs were identified. However, no common DEGs were identified between two miRNA datasets and one mRNA dataset of cSCC.

#### RNA-Seq Data Analysis of cSCC

DEGs were identified between normal & cSCC phenotypes in RNA-seq data analysis of cSCC. Two datasets of cSCC were analyzed for RNA-seq analysis as shown in **Table 1**. The **Table 3** shows the number of identified DEGs from RNA-seq datasets of cSCC. From datasets E-MTAB-5678 and GSE84293, 1693 and 349 DEGs were identified, respectively. **Supplementary Figures S17** and **S18** depict the volcano plots displaying DEGs between cSCC and normal phenotypes of E-MTAB-5678 and GSE-84293, respectively. The datasets were then compared for common genes by comparing the identified DEGs from both the analyzed datasets. As a result of this comparison, 26 common DEGs were obtained. The common DEGs are given in **Table 3**. This comparison also revealed that E-MTAB-5678 has 1667 and GSE84293 has 323 unique identified DEGs, as shown in **Figure 2**.

### Pathway Analysis

Pathway analysis of DEGs of psoriasis and cSCC datasets revealed the significant enrichment of these DEGs in various pathways. The significant pathways (at p-value <= 0.05) of all the analyzed datasets are provided in **Supplementary Table 1**. Out of these significant pathways, IL-17 signaling pathway was found common among the analyzed datasets of both psoriasis and cSCC, as given in **Table 4**.

**Table 4:** Common pathway of both the diseases. The DEGs of both the datasets of psoriasis and cSCC were enriched in IL-17 signaling pathway.

Sr. no.	Pathway	Psoriasis Datasets	cSCC Datasets
1	IL-17 signaling pathway	E-GEOD-34248 E-GEOD-41664 E-GEOD-50790 E-GEOD-13355 (lesional vs normal) E-GEOD-75343 (lesional vs normal) E-GEOD-13355 (non-lesional vs normal) E-GEOD-75343 (non-lesional vs normal) E-GEOD-67785	E-GEOD-42677 (cSCC-insitu vs normal) E-GEOD-42677 (cSCC-invasive vs normal) GSE84293

### Quantitative Systems Biology

#### Simulation of the Model

**Figure 3:**
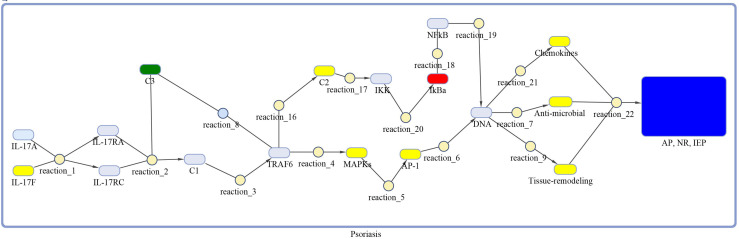
Model of IL-17 signaling pathway for psoriasis. The model is built as network of species (oval) and reactions (circle), which are connected by edges. Species represent biological entities of the pathway, and are color coded. Dark blue color shows processes of autoimmune pathology, neutrophil recruitment, and immunity to extracellular pathogens, yellow color depicts DEGs, green color shows DEG that are also acting as inhibitor, red color shows inhibitors, and sky-blue color represents genes that are part of the pathway, however, not differentially expressed. The species MAPKs includes MAPK13 and MAPK14. Chemokines includes CXCL1, CXCL2, CXCL5, CXCL8, CXCL10, CCL2, CCL7 and CCL20. The species Anti-microbial includes S100A7, S100A8, S100A9, and LCN2 genes. Tissue remodeling includes MMP1 and MMP9 genes. C1, C2, C3 and AP-1 are complexes of genes. C1 includes Hsp90 and Act1. C2 includes TAB2, TAB3 and TAK1 genes. C3 incorporates ANAPC5 and A20 genes, and AP-1 includes FOSL1 and FOS genes.

**Figure 4:**
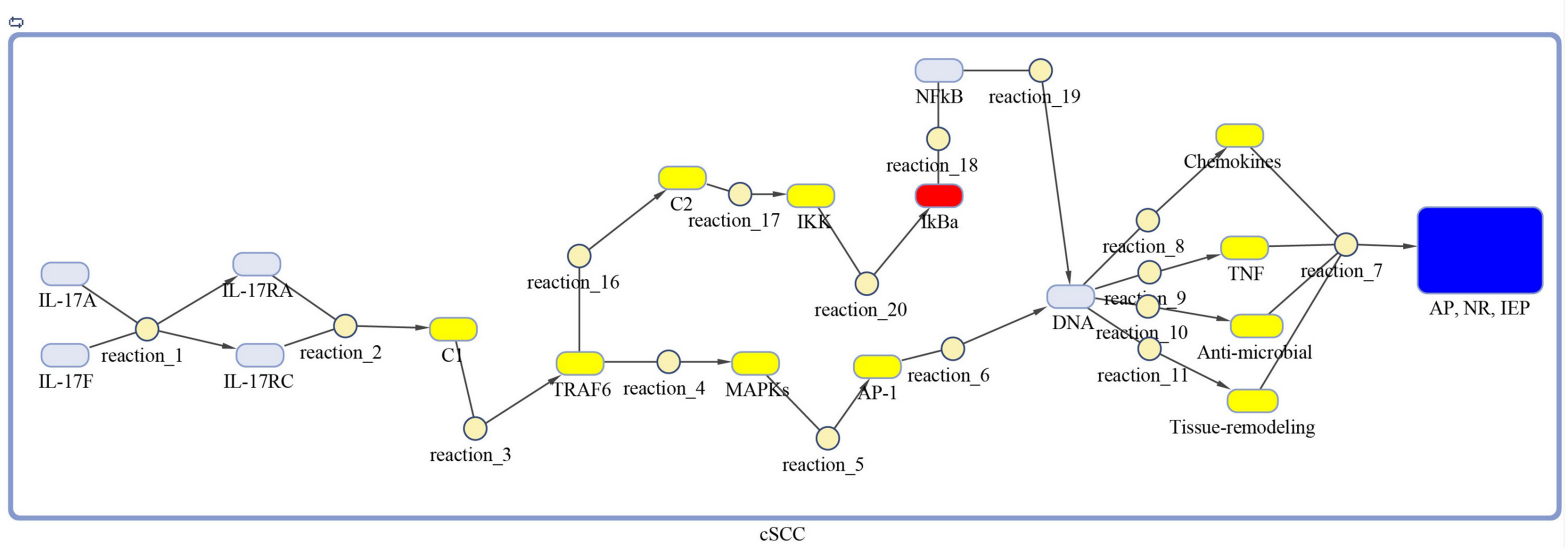
Model of IL-17 signaling pathway for cSCC. The model is built as network of species (oval) and reactions (circle), which are connected by edges. Species represent biological entities of the pathway, and are color coded. Dark blue color shows processes of autoimmune pathology, neutrophil recruitment, and immunity to extracellular pathogens, yellow color depicts DEGs, red color shows inhibitors, and sky-blue color represents genes that are part of the pathway, however, not differentially expressed. The species MAPKs includes MAPK3, MAPK9, MAPK10, MAPK11, and MAPK14. Chemokines includes CXCL1, CXCL2, CXCL8, and CCL20. The species Anti-microbial includes DEFB4A, DEFB4B, S100A7, S100A8, S100A9, and LCN2 genes. Tissue remodeling includes MMP1, MMP3, MMP9, and MMP13 genes. C1, C2, C3 and AP-1 are complexes of genes. C1 includes Hsp90 and Act1. C2 includes TAB2, TAB3 and TAK1 genes. AP-1 includes FOSL1 and JUN genes.

**Figure 5:**
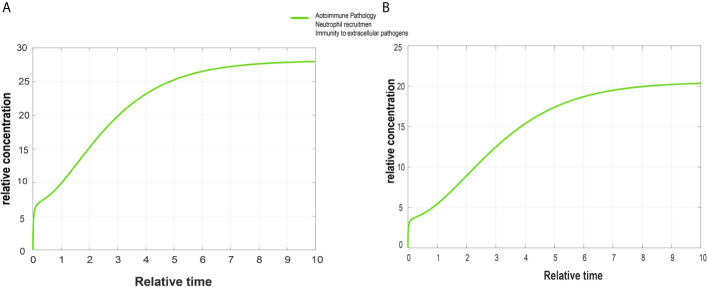
Simulated graphs of autoimmune pathology, neutrophil recruitment, and immunity to extracellular pathogens. The x-axis represents time, whereas y-axis depicts concentration. **(A)** Simulated graph of autoimmune pathology, neutrophil recruitment, and immunity to extracellular pathogens in psoriasis. In psoriasis, activation of autoimmune pathology, neutrophil recruitment, and immunity to extracellular pathogens start immediately and reach their peak value around 28 in 9.5 time units and become constant after it. **(B)** Simulated graph of autoimmune pathology, neutrophil recruitment, and immunity to extracellular pathogens in cSCC. Autoimmune pathology, neutrophil recruitment, and immunity to extracellular pathogens activate immediately in cSCC and reach their peak value around 21 in 10 time units.

The DEGs of psoriasis and cSCC were significantly enriched in the IL-17 signaling pathway. A sub-section of complete IL-17 signaling pathway was modeled in Matlab for analyzing the sensitivity of autoimmune pathology, neutrophil recruitment, and immunity to extracellular pathogens towards DEGs. These models for psoriasis and cSCC are shown in **Figures 3** and **4**, respectively. The models were simulated by inserting the average expression values (given in **Table 5**) of DEGs in psoriasis and cSCC enriched in IL-17 signaling pathway. The simulation of model unveiled the alterations in the concentrations of species over time. The **Figures 5A, B** depict simulated processes of autoimmune pathology, neutrophil recruitment, and immunity to extracellular pathogens in psoriasis and cSCC, respectively. It is cleared from **Figures 5A, B** that the rate of these biological processes increases over time in both psoriasis and cSCC. However, the rate is relatively higher in psoriasis. The peak value of these biological processes in psoriasis is around 28 in 9.5 time units, whereas it is observed to be around 21 in 10 time units in cSCC.

**Table 5:** DEGs of psoriasis and cSCC significantly enriched in IL-17 signaling pathway.

Sr. no.	Psoriasis DEGs	Average Expressions	cSCC DEGs	Average Expressions
1	CXCL2	7.143566	SRSF1	11.30402
2	S100A8	13.03884	TNF	3.775442
3	S100A9	11.57422	S100A8	10.50457
4	LCN2	10.42698	ELAVL1	7.146701
5	CCL20	7.295939	TRAF6	5.351013
6	CXCL8	7.35222	LCN2	5.506456
7	FOSL1	6.836052	MAPK11	4.413777
8	IL1B	7.299115	CXCL3	4.455207
9	CXCL1	7.507195	DEFB4A	7.363343
10	S100A7	13.38452	JUN	9.363052
11	MMP1	7.214311	DEFB4B	7.363343
12	IFNG	5.405429	IKBKG	6.876049
13	CXCL10	8.052625	TRAF3IP2	3.874313
14	MAPK14	8.921881	FOSL1	4.520605
15	CCL2	9.811309	MAPK3	7.049278
16	MMP9	8.010812	S100A9	8.624269
17	IL17D	9.42729	IL1B	3.671266
18	MAPK13	11.11599	CCL20	4.261092
19	TAB3	5.693991	MAPK10	4.192857
20	IL17A	3.848178	MAPK9	4.239504
21	CCL7	4.973044	TRAF2	5.059843
22	IL17F	5.032107	S100A7	10.17163
23	IKBKE	6.796894	JUND	3.783349
24	IL17RE	8.205539	HSP90AA1	11.51992
25	ANAPC5	7.907747	MAPK14	5.104123
26	FOS	8.704977	MMP3	4.519938
27			MAP3K7	7.460711
28			MMP13	4.249849
29			TRADD	8.777471
30			MMP9	6.08175
31			MMP1	5.822357
32			CXCL8	3.900222
33			CXCL1	4.924705
34			IKBKE	6.151091

The average expression values were calculated for microarray datasets. The DEGs of psoriasis are of lesional vs non-lesional and lesional vs normal groups. The DEGs of cSCC are of cSC-insitu vs normal and cSCC-invasive vs normal groups

#### Sensitivity Analysis

**Figure 6:**
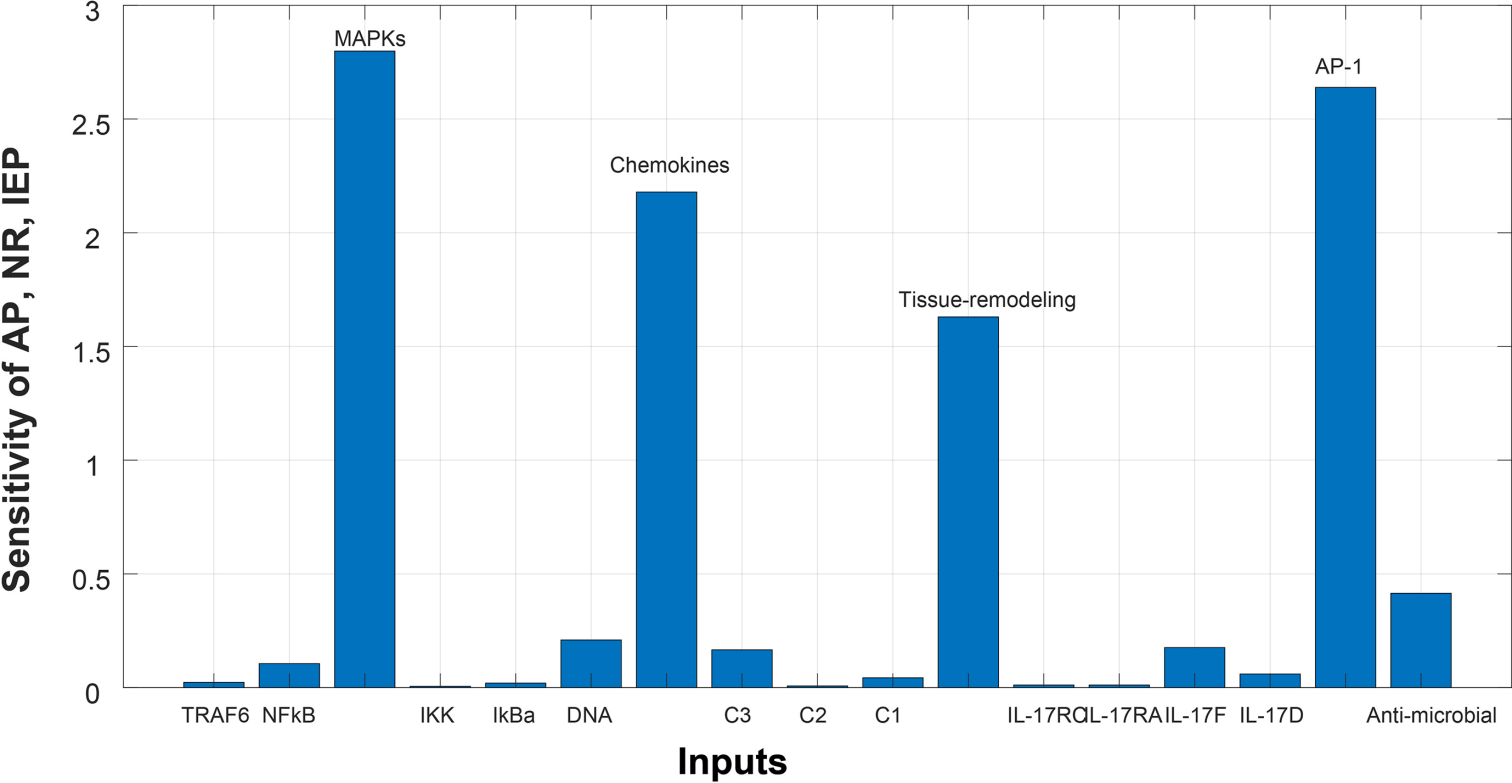
Sensitivity analysis of Autoimmune pathology (AP), neutrophil recruitment (NR), and immunity to extracellular pathogens (IEP) in psoriasis. The x-axis represents input species (genes), whereas y-axis depicts sensitivity of AP, NR and IEP. The high influence of MAPKs, chemokines, tissue remodeling genes, and AP-1 is observed in AP, NR and IEP. It indicates that AP, NR and IEP are highly sensitive towards these genes, having sensitivity values greater than 1.5.

**Figure 7:**
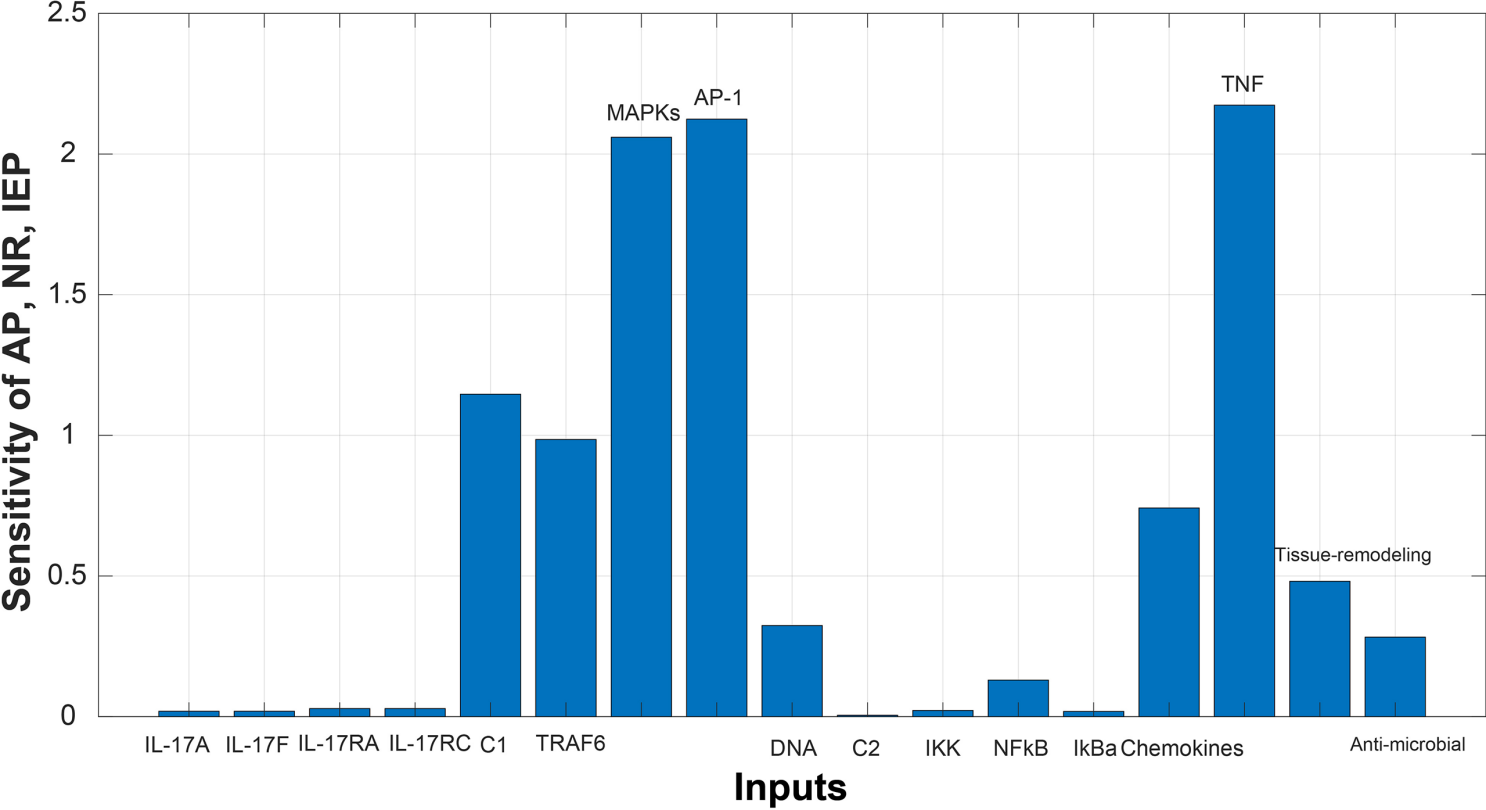
Sensitivity analysis of Autoimmune pathology (AP), neutrophil recruitment (NR), and immunity to extracellular pathogens (IEP) in cSCC. The x-axis represents input species (genes), whereas y-axis depicts sensitivity of AP, NR and IEP. The high influence of MAPKs, AP-1, and TNF is observed in AP, NR and IEP. It indicates that AP, NR and IEP are highly sensitive towards these genes, having sensitivity values greater than 1.5.

Sensitivity analysis was performed to study the impact of the species and reactions of the model on biological processes of autoimmune pathology, neutrophil recruitment, and immunity to extracellular pathogens. The **Figures 6** and **7** depict the sensitivity analysis of these biological processes in psoriasis and cSCC, respectively. It is cleared from **Figure 6** that the biological processes are highly sensitive towards MAPKs, chemokines, tissue remodeling genes, and AP-1 (FOSL1 and FOS genes) in psoriasis, having sensitivity values greater than 1.5. Whereas in cSCC, these biological processes are highly sensitive towards MAPKs, AP-1 (FOSL1 and JUN genes), and TNF genes, having sensitivity values greater than 1.5 as shown in **Figure 7**.

## Discussion

Psoriasis is one of the most common and chronic skin diseases (1). It is characterized by rough scales on the skin. It could appear on any part of the body and it affects both children and adults (1). About 2-3% individuals are affected by psoriasis worldwide (2), and the incidence rate of psoriasis is also observed to increase with time (3). Association of psoriasis with other comorbidities, like cardiovascular diseases, diabetes, and metabolic syndrome, has been observed (12). Studies have also revealed the possibility of association of psoriasis with skin cancers, particularly non-melanoma skin cancers (NMSC), which include basal cell carcinoma (BCC) and cutaneous Squamous Cell Carcinoma (cSCC) (12, 16). Hence, the second analyzed disease in this study is cSCC. It is the second most prevalent nonmelanoma skin cancer (19, 20) with potential to metastasize and recur in some cases (19). Moreover, the incidence rate of cSCC is also increasing annually (19, 20). Therefore, there exists a dire need to analyze both the diseases at the molecular level to propose potential biomarkers and common therapeutic targets.

This study was designed to interpret and compare the high-throughput data of psoriasis and cSCC, aiming to reveal potential biomarkers, common therapeutic targets, and the association between the two diseases. Microarray and RNA-seq data analyses were performed on the datasets of psoriasis and cSCC (given in **Table 1**) to reveal the common and uncommon patterns. After performing differential expression analysis, pathway analysis was performed to analyze the disrupted pathways and their possible relation with the disease. The common pathway between the diseases was selected for further analysis. Afterwards, quantitative systems biology was implemented, by using SimBiology application in Matlab (30), to analyze the results at a holistic level. The differential expression analysis of both the analyzed diseases resulted in the identification of DEGs. Top 10 DEGs (along with p-value and *Log_2_FC*) from all the analyzed datasets are provided in **Supplementary Tables S1–S18**. The identified number of DEGs from microarray data analysis are given in **Table 2**. The comparison of microarray datasets of psoriasis resulted in one common DEG, named S100A8. The significant role of S100A8 in prognosis of psoriasis is also illustrated in the literature (48). The overexpression of S100A8 causes keratinocyte proliferation and angiogenesis in psoriatic skin (47, 49). The comparison of microarray datasets of cSCC resulted in no common gene. The number of identified DEGs from RNA-seq data analysis is given in **Table 3**. The comparison of RNA-seq datasets resulted in 16 and 26 common DEGs for psoriasis and cSCC, respectively (given in **Table 3**). These common DEGs could be proposed as potential biomarkers of the diseases. The overall comparative analysis of identified DEGs from all the analyzed datasets of both the diseases resulted in common DEGs between the two diseases, as given in **Table 6**. These DEGs may represent the potential biomarkers or therapeutic targets of psoriasis and cSCC.

**Table 6:** Potential biomarkers and therapeutic targets for psoriasis and cSCC.

Sr. no.	Potential Therapeutic Targets	Psoriasis Datasets	cSCC Datasets
1	S100A8	E-GEOD-34248 E-GEOD-41664 E-GEOD-50790 E-GEOD-67785 E-GEOD- 13355 (lesional vs normal) E-GEOD-75343 (lesional vs normal) E-GEOD-13355 (lesional vs non-lesional) E-GEOD-75343 (lesional vs non-lesional)	E-GEOD-42677 (cSCC-insitu vs normal) E-GEOD-42677 (cSCC-invasive vs normal) E-MTAB-5678 GSE84293
2	TMPRSS11D	E-GEOD-34248 E-GEOD-41664 E-GEOD-41745 E-GEOD-50790 E-GEOD-67785 E-GEOD- 13355 (lesional vs normal) E-GEOD-75343 (lesional vs normal) E-GEOD-13355 (lesional vs non-lesional) E-GEOD-75343 (lesional vs non-lesional)	E-GEOD-42677 (cSCC-insitu vs normal) E-GEOD-42677 (cSCC-invasive vs normal) GSE84293
3	SERPINB4		
4	ADH1B	E-GEOD-34248 E-GEOD-41664 E-GEOD-50790 E-GEOD- 13355 (lesional vs normal) E-GEOD-75343 (lesional vs normal) E-GEOD-13355 (lesional vs non-lesional) E-GEOD-75343 (lesional vs non-lesional)	E-GEOD-42677 (cSCC-insitu vs normal) E-GEOD-42677 (cSCC-invasive vs normal) E-MTAB-5678 GSE84293
5	TYMP	E-GEOD-34248 E-GEOD-41664 E-GEOD-50790 E-GEOD-67785 E-GEOD- 13355 (lesional vs normal) E-GEOD-75343 (lesional vs normal) E-GEOD-13355 (lesional vs non-lesional) E-GEOD-75343 (lesional vs non-lesional)	E-GEOD-42677 (cSCC-insitu vs normal) E-GEOD-42677 (cSCC-invasive vs normal) E-MTAB-5678
6	KLK6		
7	IFI6		
8	SPRR1A		
9	OAS2	E-GEOD-34248 E-GEOD-41664 E-GEOD-50790 E-GEOD-67785 E-GEOD- 13355 (lesional vs normal) E-GEOD-75343 (lesional vs normal) E-GEOD-13355 (lesional vs non-lesional) E-GEOD-75343 (lesional vs non-lesional)	E-GEOD-42677 (cSCC-insitu vs normal) E-GEOD-42677 (cSCC-invasive vs normal) GSE84293
10	AKR1B10		
11	TCN1		
12	KRT16		
13	DSC2		
14	S100A9		
15	CCL27		
16	KCNJ15		
17	PI3	E-GEOD-34248 E-GEOD-41664 E-GEOD-41745 E-GEOD-50790 E-GEOD-67785 E-GEOD- 13355 (lesional vs normal) E-GEOD-75343 (lesional vs normal) E-GEOD-13355 (lesional vs non-lesional) E-GEOD-75343 (lesional vs non-lesional)	E-GEOD-42677 (cSCC-insitu vs normal) E-GEOD-42677 (cSCC-invasive vs normal)
18	ATP12A		
19	IFI27		
20	S100A12		
21	LCN2		
22			
23	OASL	E-GEOD-34248 E-GEOD-41664 E-GEOD-50790 E-GEOD-67785 E-GEOD- 13355 (lesional vs normal) E-GEOD-75343 (lesional vs normal) E-GEOD-13355 (lesional vs non-lesional) E-GEOD-75343 (lesional vs non-lesional)	E-GEOD-42677 (cSCC-insitu vs normal) E-GEOD-42677 (cSCC-invasive vs normal)
24	ISG15		
25	CRAT		
26	STAT1		
27	KYNU		
28	CD24		
29	UPP1		
30	SERPINB3		
31	PNP		
32	MX1		
33	CCL20		

Pathway analysis of DEGs of psoriasis and cSCC revealed the enrichment of these DEGs in various pathways (provided in **Supplementary Table 1**). The current study has proposed one common significant pathway, IL-17 signaling pathway, based on the enrichment of DEGs of both the diseases in that pathway (given in **Table 4**). Genome-wide association and other genetic studies have clearly linked multiple IL-17–related genes to psoriasis pathogenesis. Psoriasis is one of the most typical IL-17A-driven human diseases (50). The expression of various IL-17 signaling pathway genes elevated in psoriatic lesional tissue compared with non-lesional tissue (51). The significance of IL-17 signaling pathway in the prognosis of cSCC is also illustrated in the literature, as the disrupted IL-17 pathway promotes tumor development by activating cytokines production (52).

IL-17 signaling pathway was selected for further analysis, as it was common in both psoriasis and cSCC datasets. IL-17 signaling play a critical role in tumor microenvironment by facilitating tumor formation and metastasis (53). Quantitative systems biology was implemented to analyze the results at a holistic level. A sub-section of complete IL-17 pathway was modeled by using SimBiology application in Matlab (30). The average expressions values of DEGs that were present in the pathway were calculated for microarray datasets of psoriasis and cSCC, as given in **Table 5**. The model was then simulated by adding the average expression values to the species (genes) as initial state values. The biological processes being analyzed in the model were autoimmune pathology, neutrophil recruitment, and immunity to extracellular pathogens. Autoimmunity is defined as when the immune system of the body becomes hyperactive and acts against self-antigen, thus, resulting in tissue damage (54). Cancer is often associated with autoimmunity. Autoimmune disorders like scleroderma and myositis might increase the risk of cancer (55). Neutrophils are the type of leukocytes that protect the body in state of infections and inflammation (56). Clinical evidence has revealed that tumor associated neutrophils (TANs) might play a critical role in tumor progression. Adaptive immune system of the host becomes activated by exhibiting particular responses to extracellular pathogens (57). The clinical evidence has suggested that abnormal adaptive immunity lead to tumor cell proliferation by inducing immunosuppression (58).

The **Figures 5A, B** depict simulated processes of autoimmune pathology, neutrophil recruitment, and immunity to extracellular pathogens in psoriasis and cSCC, respectively. It is cleared from **Figures 5A, B** that the rate of these biological processes increases over time in both psoriasis and cSCC. However, the rate is relatively higher in psoriasis. The peak value of these biological processes in psoriasis is around 28 in 9.5 time units, whereas it is observed to be around 21 in 10 time units in cSCC.

After simulation of the model, sensitivity analysis was performed to analyze the response of the biological processes to model quantities (DEGs and genes). The sensitivity analysis revealed that autoimmune pathology, neutrophil recruitment, and immunity to extracellular pathogens are highly sensitive towards MAPKs, chemokines, tissue remodeling genes, and AP-1 (FOSL1 and FOS genes) in psoriasis, having sensitivity values greater than 1.5 **Figure 6**. It indicates that the slight changes in these genes profoundly affect the biological processes. These results are also consistent with the literature. Disrupted signaling pathways are associated with the complex pathogenesis of psoriasis, particularly, the possible pathogenic role of disrupted MAPK signaling in the development of psoriasis (59, 60). Chemokines and chemokine receptors are involved in the pathogenesis of psoriasis by facilitating the trafficking of T-cells in psoriasis (61). Therefore, targeting chemokines in psoriasis would result in therapeutic effect and improved treatment for psoriasis (61, 62). Tissue remodeling genes (MMPs) contribute to the pathogenesis of psoriasis by stimulating angiogenesis and intrusion of immune cells in the dermal tissue (63). AP-1 (the transcription factor activator protein 1) functions in cell proliferation. However, the binding ability of AP-1 is highly damaged in psoriatic lesional skin (64), thus genes for AP-1 are significant in pathogenesis of psoriasis and proposed as candidate target genes (65).

Whereas the sensitivity analysis of cSCC revealed that the biological processes are highly sensitive towards MAPKs, AP-1 (FOSL1 and JUN genes), and TNF genes, having sensitivity values greater than 1.5 as shown in **Figure 7**. The slight changes in these genes profoundly affect the biological processes. These results are also consistent with the literature. Dysregulated MAPK signaling by high levels of HMG-1 protein stimulates metastasis in cSCC (66). Dysregulation in the expression of the genes for AP-1 (the transcription factor activator protein 1) promote cancer development as AP-1 factors are involved in keratinocyte proliferation of skin (67). TNF(Tumor necrosis factor) is a proinflammatory cytokine that mainly functions in cell proliferation, differentiation, and death, hence plays an important role in carcinogenesis (68).

Sensitivity analysis of the biological processes of both psoriasis and cSCC revealed MAPKs and genes for AP-1 as significant genes. Slight change in these genes would greatly impact autoimmune pathology, neutrophil recruitment, and immunity to extracellular pathogens. Therefore, consistent with the discussion we propose that MAPKs (MAPK13 and MAPK14) and genes for AP-1 (FOSL1 and FOS) identified from sensitivity analysis of psoriasis are significant genes that might lead psoriasis towards cancer.

## Conclusion

In the current study, the integrated data analysis of microarray and RNA-seq was performed to reveal common patterns between psoriasis and cSCC. The differential expression analysis of both the diseases resulted in common DEGs, which are proposed as potential biomarkers and therapeutic targets for psoriasis and cSCC. IL-17 signaling pathway is identified as common significant pathway as DEGs of both the diseases were enriched in that pathway. IL-17 was further studied, and sensitivity analysis was performed that resulted in the identification of significant genes in psoriasis, that might lead psoriasis towards cancer. These significant genes are MAPKs (MAPK13 and MAPK14) and genes for AP-1 (FOSL1 and FOS). These genes should be further investigated at experimental level for validating their roles in the transfer of psoriatic condition to cSCC.

## Data Availability Statement

The datasets presented in this study can be found in online repositories. The names of the repository/repositories and accession number(s) can be found in the article/**Supplementary Material**.

## Author Contributions

SA and RP conceived and designed the study. SA conducted the analysis and wrote the manuscript. All authors contributed to the article and approved the submitted version.

## Funding

National University of Sciences and Technology, Islamabad paid the publication fee for this article.

## Conflict of Interest

The authors declare that the research was conducted in the absence of any commercial or financial relationships that could be construed as a potential conflict of interest.
