# Supplementary material for: A Computational Systems Analyses to Identify Biomarkers and Mechanistic Link in Psoriasis and Cutaneous Squamous Cell Carcinoma
Source: Front Immunol. 2021 Jun 18;12:662528. doi: 10.3389/fimmu.2021.662528 (PMC8276676; doi:10.3389/fimmu.2021.662528)
Supplement: Supplementary file 1 [file DataSheet_1.pdf]

Supplementary Files

May 18, 2021

1 Volcano plots

The log 2 transformed fold changes are plotted on x-axis while the y-axis depicts the -log 10 transformed p-values. Each dot on the plot is a gene. The dots of the plot are color coded. Gray color of the dots indicates that genes have not crossed both the set thresholds of p-value and fold change. The green dots have only passed the fold change threshold and the blue dots have only passed the p-value threshold. The red dots are the significant ones, as they have crossed both the thresholds of significance & fold change. These red dots are the identified DEGs. The positive and negative values of fold change indicate that genes are up-regulated and down-regulated, respectively.

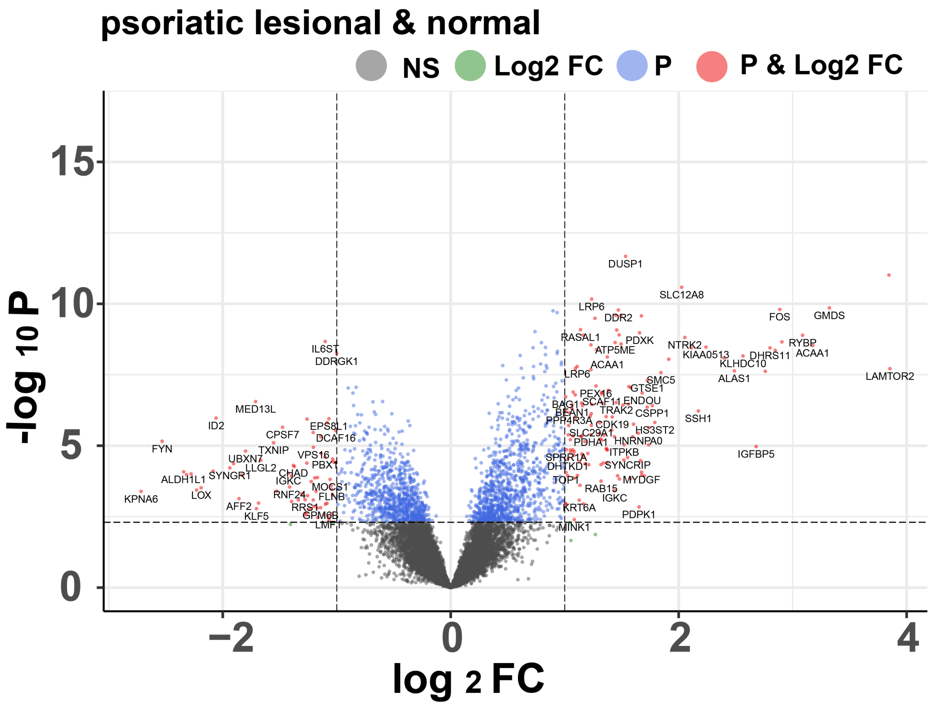

Figure (1) Volcano plot E-GEOD-75343 displaying DEGs between psoriatic lesional & normal phenotypes.

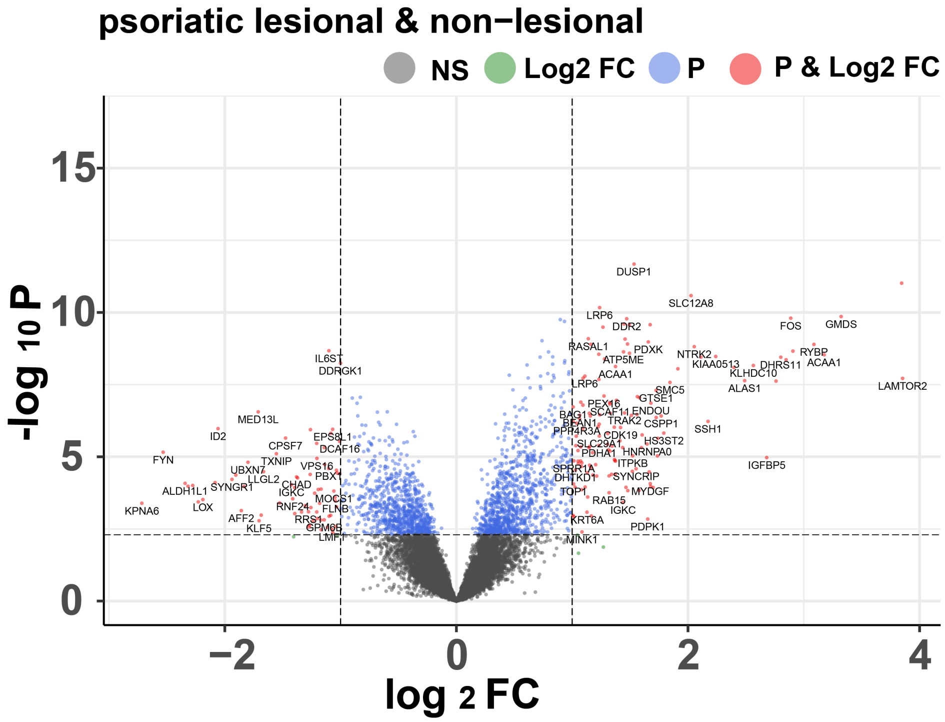

Figure (2) Volcano plot of E-GEOD-75343 displaying DEGs between psoriatic lesional & non-lesional phenotypes.

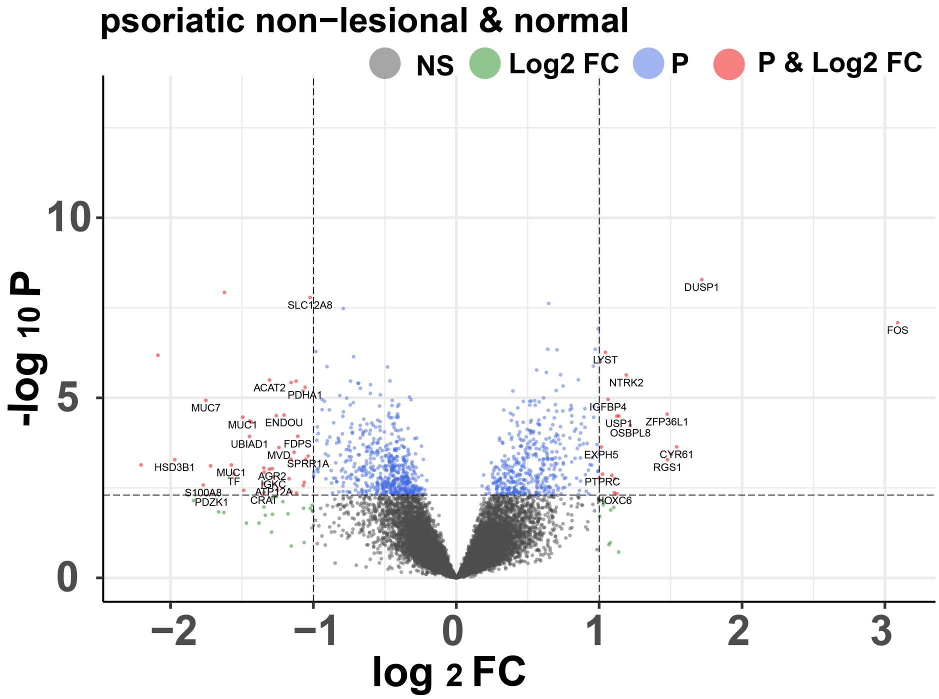

Figure (3) Volcano plot of E-GEOD-75343 displaying DEGs between psoriatic non-lesional & normal phenotypes.

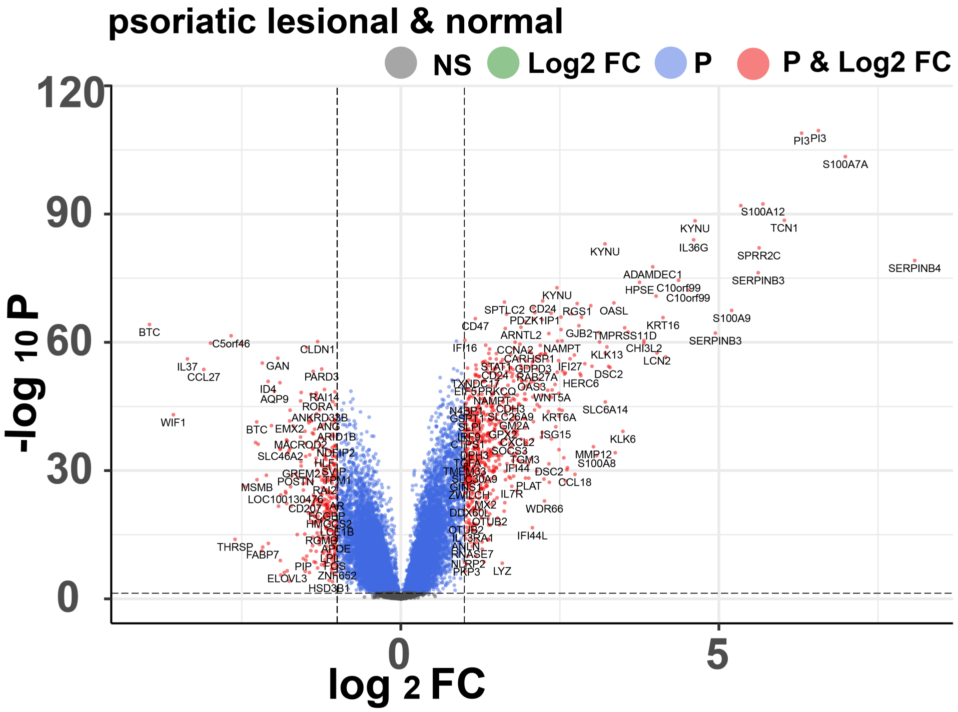

Figure (4) Volcano plot of E-GEOD-13355 displaying DEGs between psoriatic lesional & normal phenotypes.

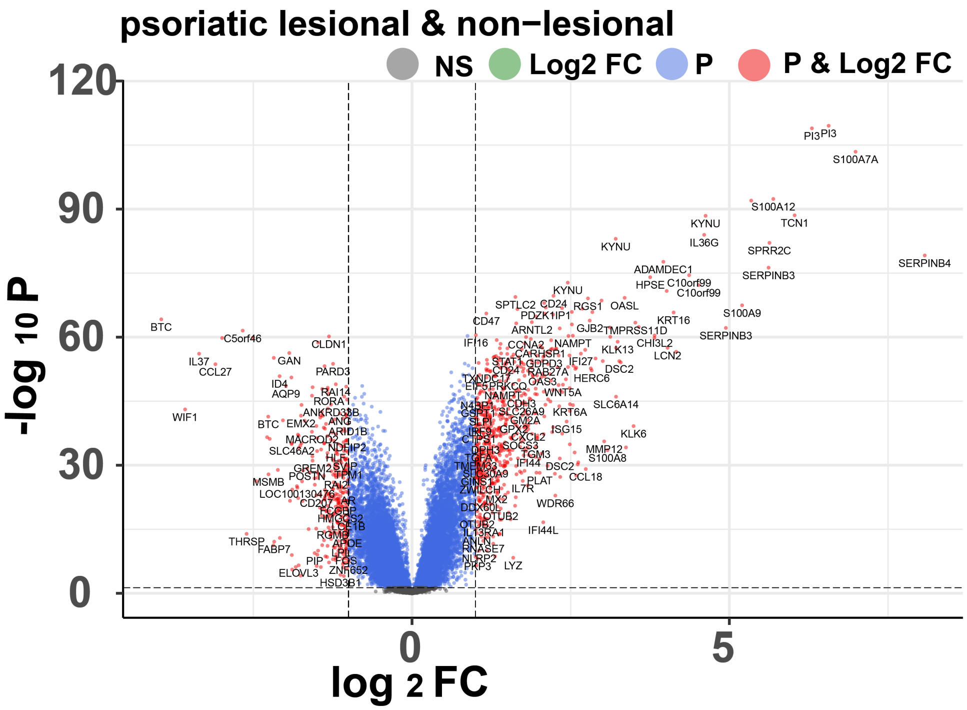

Figure (5) Volcano plot of E-GEOD-13355 displaying DEGs between psoriatic lesional & non-lesional phenotypes.

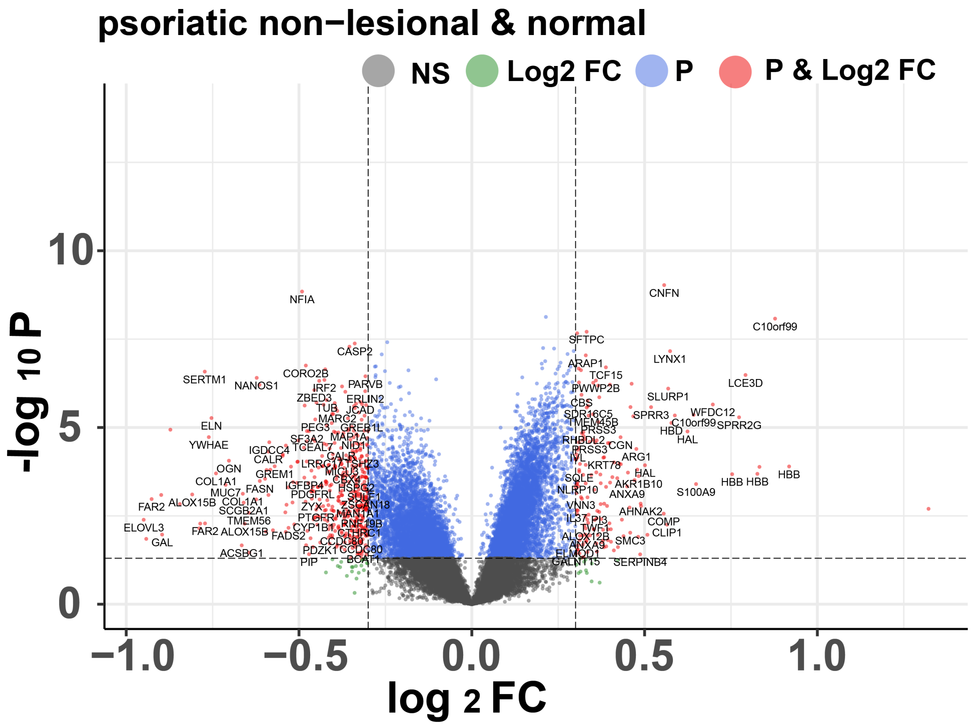

Figure (6) Volcano plot of E-GEOD-13355 displaying DEGs between psoriatic non-lesional & normal phenotypes.

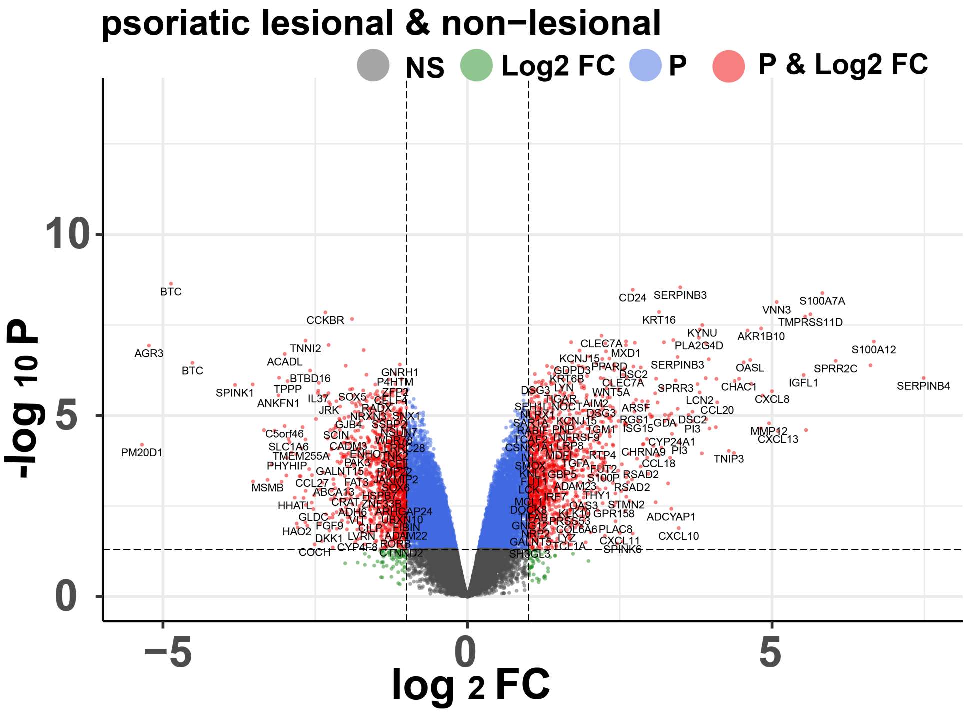

Figure (7) Volcano plot E-GEOD-50790 displaying DEGs between psoriatic lesional & non-lesional phenotypes.

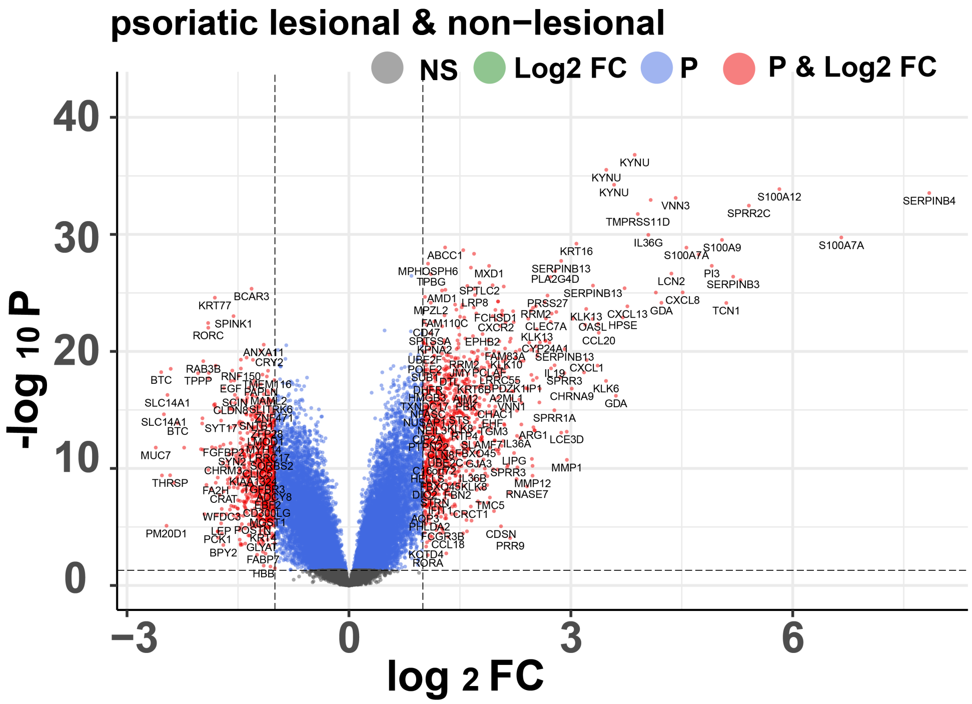

Figure (8) Volcano plot of E-GEOD-41664 displaying DEGs between psoriatic lesional & non-lesional phenotypes.

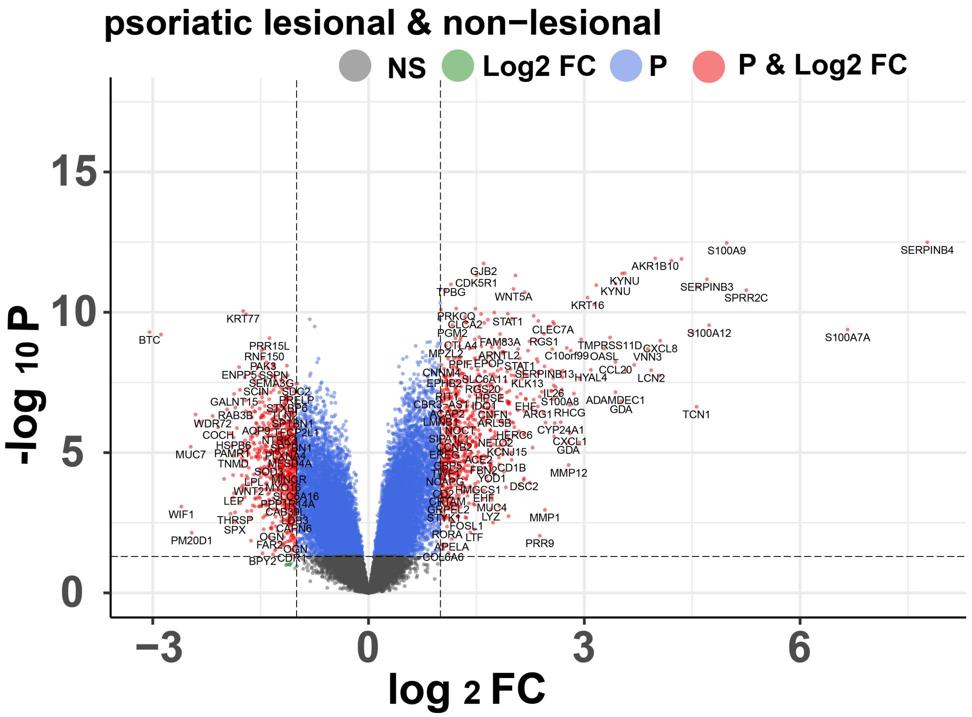

Figure (9) Volcano plot of E-GEOD-34248 displaying DEGs between psoriatic lesional & non-lesional phenotypes.

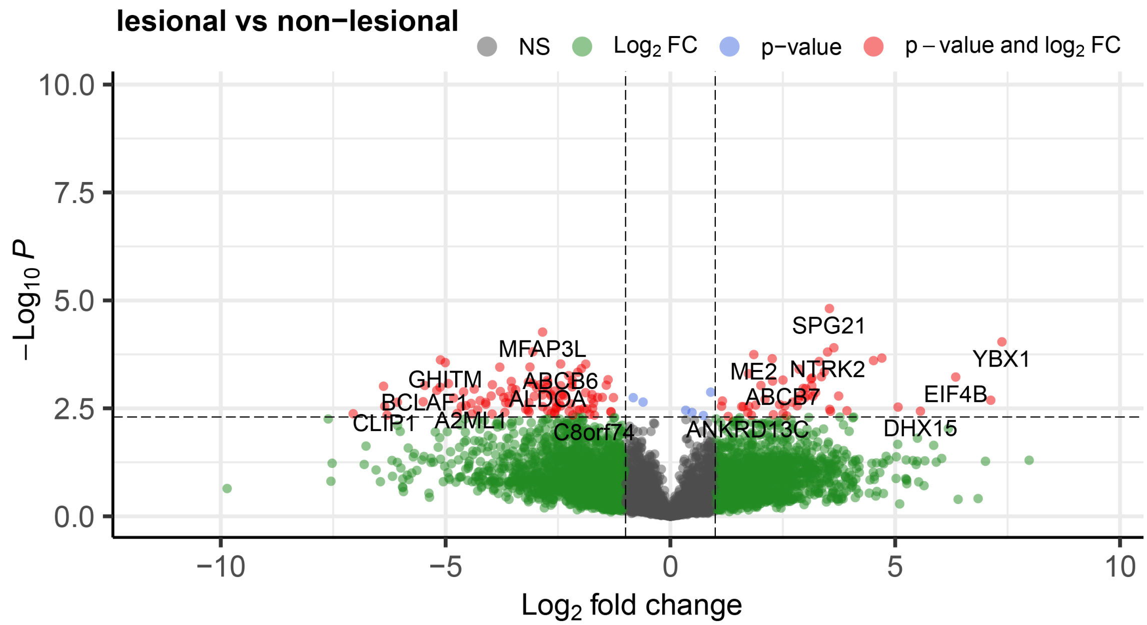

Figure (10) Volcano plot of E-GEOD-41745 displaying DEGs between psoriatic lesional & non-lesional phenotypes.

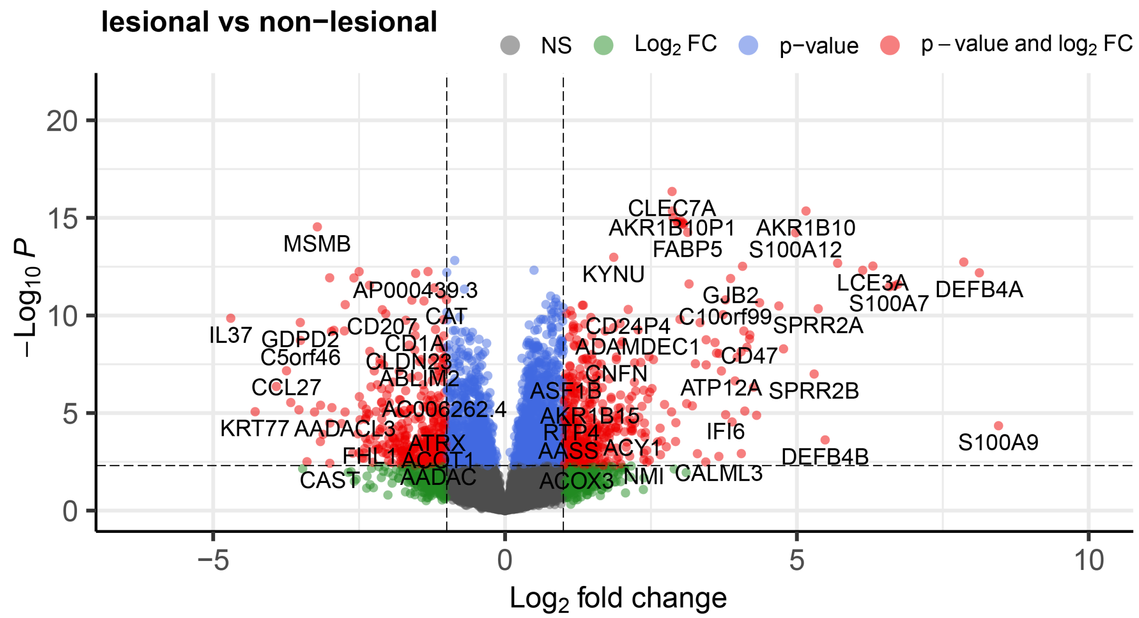

Figure (11) Volcano plot of E-GEOD-67785 displaying DEGs between psoriatic lesional & non-lesional phenotypes.

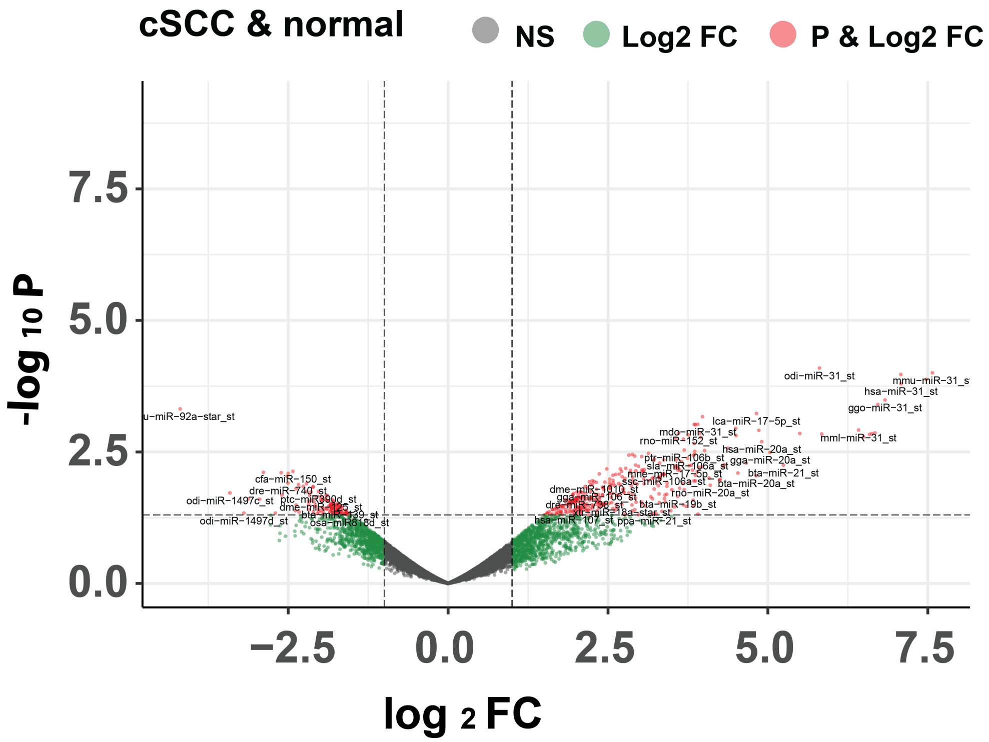

Figure (12) Volcano plot E-GEOD-32868 displaying differentially expressed miRNAs between cSCC & normal phenotypes.

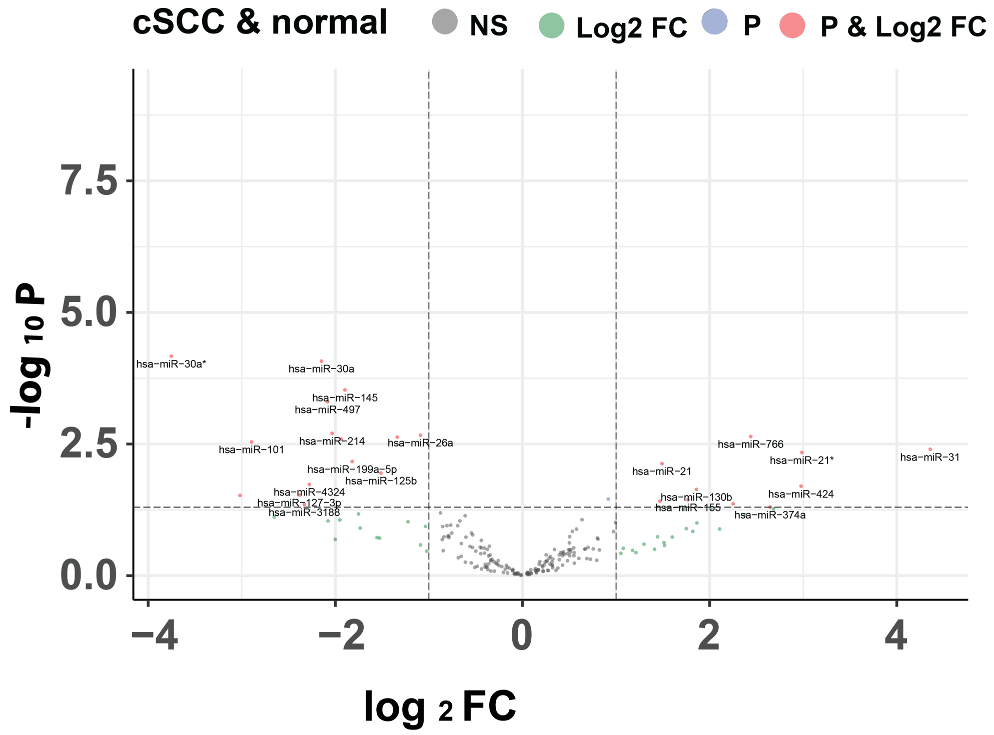

Figure (13) Volcano plot of E-GEOD-34536 displaying differentially expressed miRNAs between cSCC & normal phenotypes.

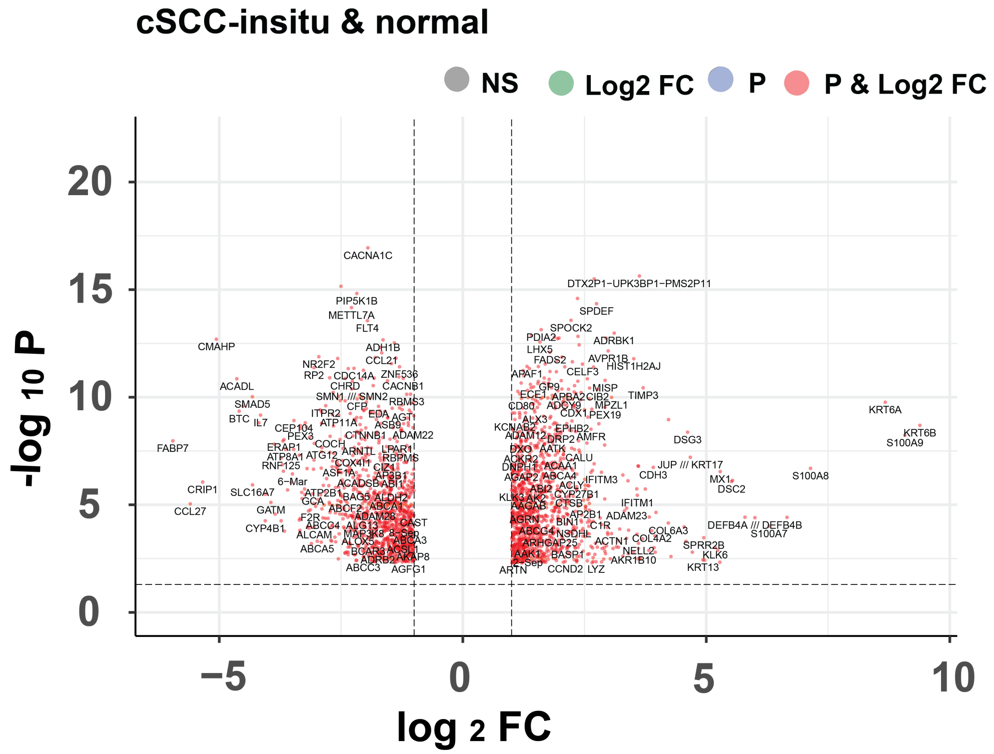

Figure (14) Volcano plot of E-GEOD-42677 displaying DEGs between cSCC-in situ & normal phenotypes.

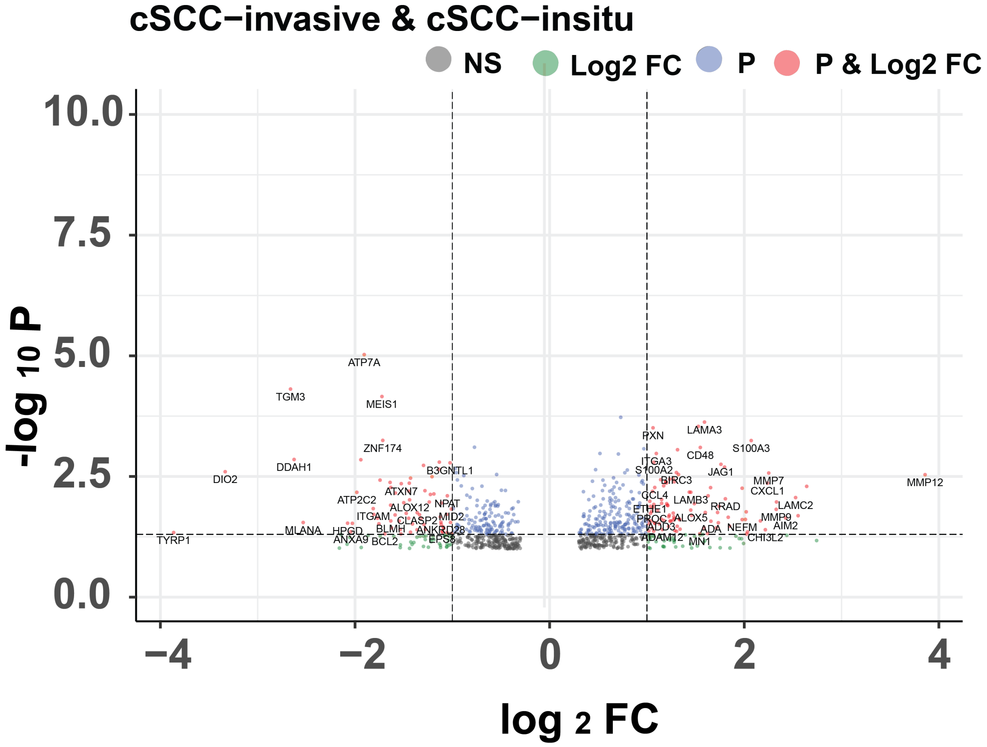

Figure (15) Volcano plot of E-GEOD-42677 displaying DEGs between cSCC-invasive & cSCC-in situ phenotypes.

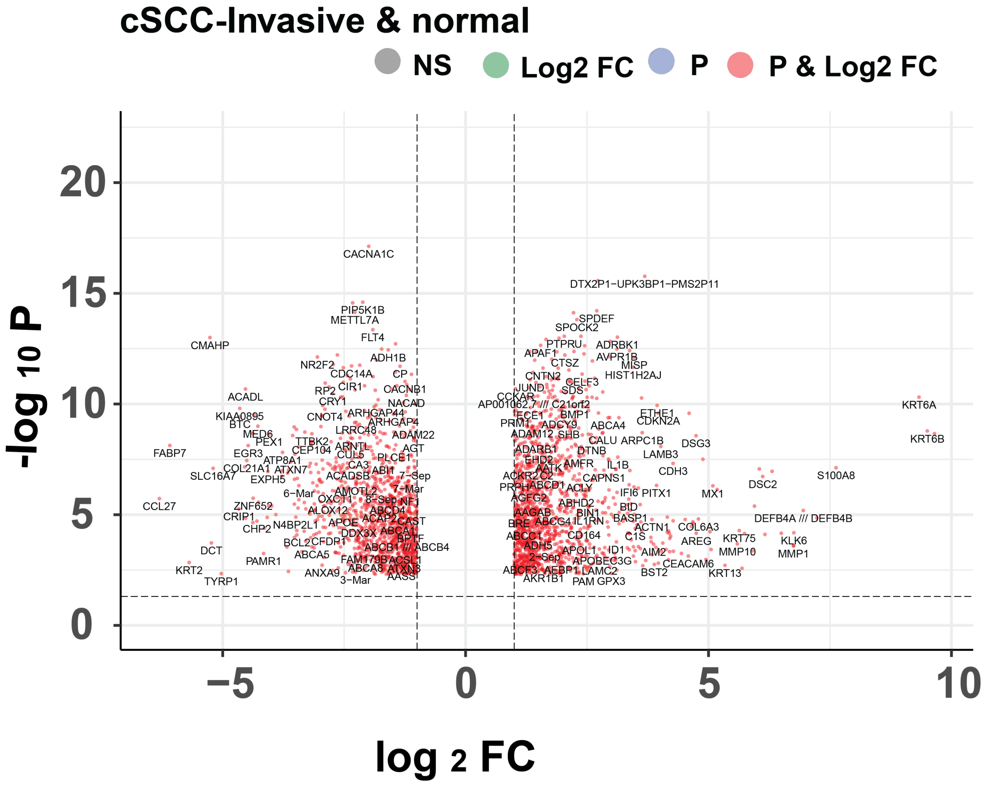

Figure (16) Volcano plot of E-GEOD-42677 displaying DEGs between cSCC-invasive & normal phenotypes.

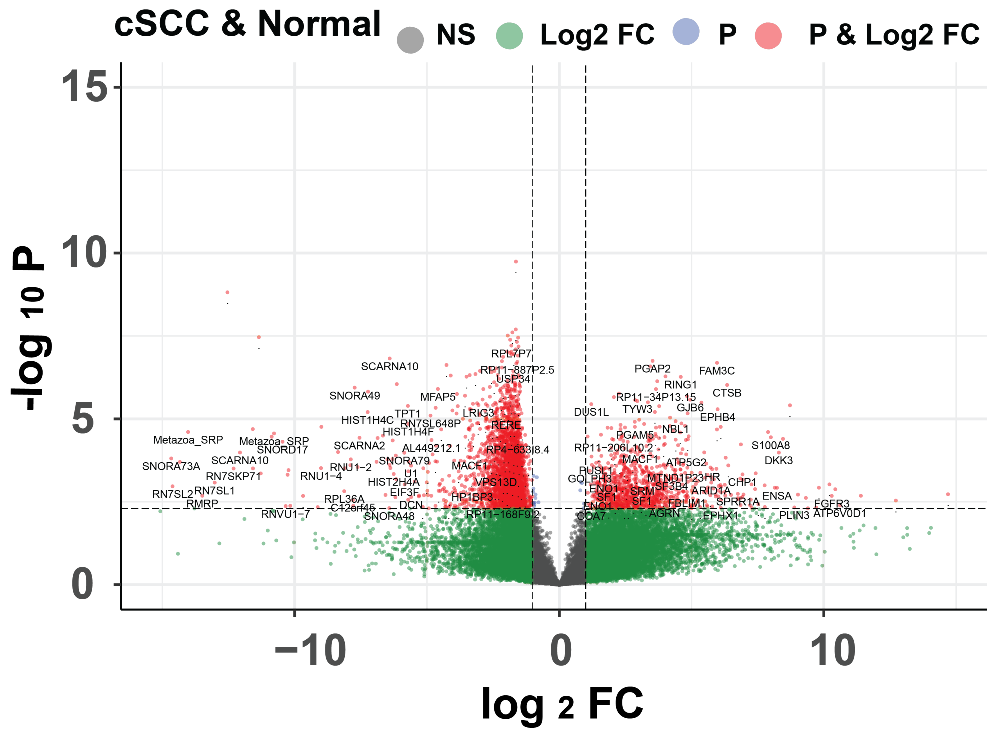

Figure (17) Volcano plot of E-MTAB-5678 displaying DEGs between cSCC & normal phenotypes.

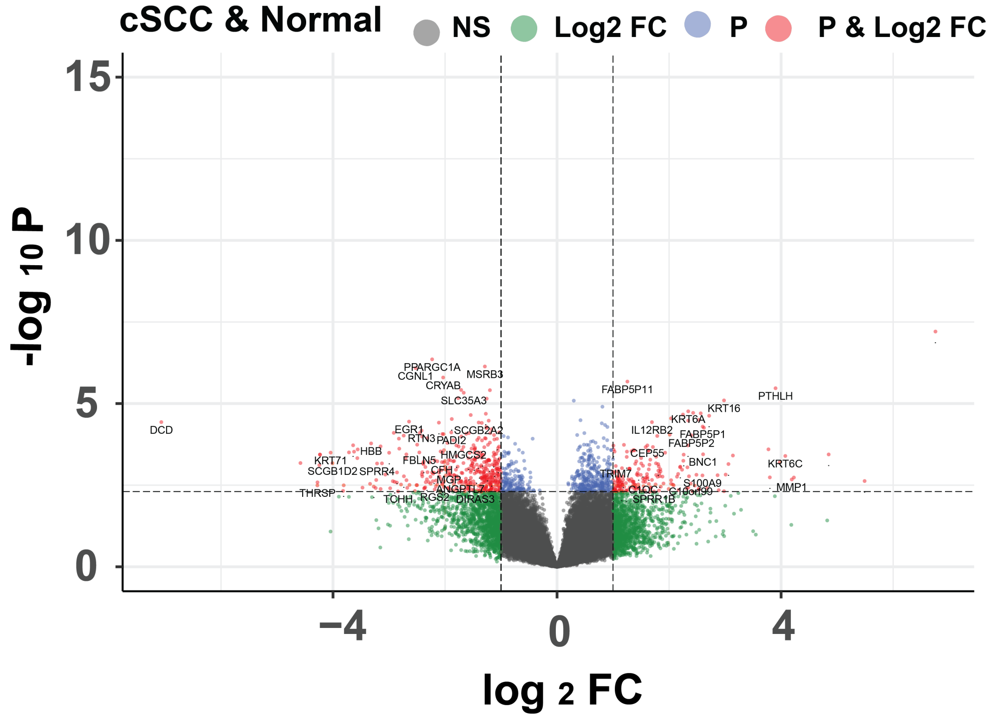

Figure (18) Volcano plot of GSE-84293 displaying DEGs between cSCC & normal phenotypes.

2 Tables

Table (1) Top 10 DEGs of lesional & normal group of E-GEOD-75343 at p-value <= 0.005 & log2(FC) <= -1 | log2(FC) >= 1

Sr. no.	SYMBOL	log2(FC)	p-value
1	ALOX12B	1.53435	2.11E-12
2	TCN1	3.84502	9.68E-12
3	IFI27	2.0267	2.61E-11
4	SCO2	1.23702	6.81E-11
5	S100A12	3.32266	1.39E-10
6	AKR1B10	2.88772	1.58E-10
7	ARNTL2	1.4709	1.66E-10
8	TNFRSF21	1.44809	2.52E-10
9	PDZK1IP1	1.49361	2.65E-10
10	OASL	1.67339	2.66E-10

Table (2) Top 10 DEGs of lesional & non-lesional group of E-GEOD-75343 at p-value <= 0.005 & log2(FC) <= -1 | log2(FC) >= 1

Sr. no.	SYMBOL	log2(FC)	p-value
1	ALOX12B	1.53435	2.11E-12
2	TCN1	3.84502	9.68E-12
3	IFI27	2.0267	2.61E-11
4	SCO2	1.23702	6.81E-11
5	S100A12	3.32266	1.39E-10
6	AKR1B10	2.88772	1.58E-10
7	ARNTL2	1.4709	1.66E-10
8	TNFRSF21	1.44809	2.52E-10
9	PDZK1IP1	1.49361	2.65E-10
10	OASL	1.67339	2.66E-10

Table (3) Top 10 DEGs of non-lesional & normal group of E-GEOD-75343 at p-value <= 0.005 & log2(FC) <= -1 | log2(FC) >= 1

**Sr. no.**	**SYMBOL**	**log2(FC)**	**p-value**
1	DUSP1	1.71787	5.21E-09
2	SLC12A8	-1.0232	1.63E-08
3	FOS	3.08895	8.22E-08
4	LYST	1.04401	5.46E-07
5	NTRK2	1.18951	2.33E-06
6	ACAT2	-1.3069	3.21E-06
7	LPIN1	-1.1212	3.45E-06
8	PDHA1	-1.0582	5.09E-06
9	APOE	-1.0726	6.51E-06
10	IGFBP4	1.06235	1.11E-05

Table (4) Top 10 DEGs of E-GEOD-50790 at p-value <= 0.005 & log2(FC) <= -1 | log2(FC) >= 1

**Sr. no.**	**SYMBOL**	**log2(FC)**	**p-value**
1	BTC	-4.8676	2.28E-09
2	SERPINB3	3.49299	2.88E-09
3	CD24	2.71244	3.35E-09
4	S100A7A	5.82583	4.15E-09
5	VNN3	5.07597	7.31E-09
6	KRT16	3.14449	1.38E-08
7	CCKBR	-2.334	1.41E-08
8	TMPRSS11D	5.62984	1.59E-08
9	SPRR3	5.54433	1.85E-08
10	KYNU	3.85464	3.17E-08

Table (5) Top 10 DEGs of E-GEOD-41664 at p-value <= 0.005 & log2(FC) <= -1 | log2(FC) >= 1

**Sr. no.**	**SYMBOL**	**log2(FC)**	**p-value**
1	KYNU	3.86325	1.59E-37
2	S100A12	5.81938	1.36E-34
3	SERPINB4	7.84543	2.93E-34
4	VNN3	4.41703	7.65E-34
5	AKR1B10	4.07947	1.14E-33
6	SPRR2C	5.40684	3.47E-33
7	TMPRSS11D	3.90495	1.82E-32
8	IL36G	4.04769	1.10E-30
9	S100A7A	6.65677	1.84E-30
10	S100A9	5.04348	2.96E-30

Table (6) Top 10 DEGs of E-GEOD-34248 at p-value <= 0.005 & log2(FC) <= -1 | log2(FC) >= 1

**Sr. no.**	**SYMBOL**	**log2(FC)**	**p-value**
1	SERPINB4	7.76828	3.21E-13
2	S100A9	4.98046	3.36E-13
3	AKR1B10	3.98652	1.20E-12
4	PI3	4.35148	1.25E-12
5	GJB2	1.60051	1.82E-12
6	KYNU	3.56207	4.05E-12
7	IL36G	3.52608	4.14E-12
8	CDK5R1	1.50036	4.76E-12
9	SERPINB3	4.70477	6.68E-12
10	TPBG	1.14603	1.01E-11

Table (7) Top 10 DEGs of lesional & normal group of E-GEOD-13355 at p-value <= 0.005 & log2(FC) <= -1 | log2(FC) >= 1:

**Sr. no.**	**SYMBOL**	**log2(FC)**	**p-value**
1	PI3	6.56373	3.02E-110
2	S100A7A	6.98774	3.64E-104
3	S100A12	5.69177	4.23E-93
4	AKR1B10	5.34403	1.02E-92
5	TCN1	6.02815	2.84E-89
6	KYNU	4.62501	3.69E-89
7	IL36G	4.60309	1.06E-84
8	SPRR2C (pseudogene)	5.63155	8.01E-83
9	SERPINB4	8.07778	7.20E-80
10	ADAMDEC1	3.9596	2.17E-78

Table (8) Top 10 DEGs of lesional & non-lesional group of E-GEOD-13355 at p-value <= 0.005 & log2(FC) <= -1 | log2(FC) >= 1

**Sr. no.**	**SYMBOL**	**log2(FC)**	**p-value**
1	PI3	6.56373	3.02E-110
2	S100A7A	6.98774	3.64E-104
3	S100A12	5.69177	4.23E-93
4	AKR1B10	5.34403	1.02E-92
5	TCN1	6.02815	2.84E-89
6	KYNU	4.62501	3.69E-89
7	IL36G	4.60309	1.06E-84
8	SPRR2C (pseudogene)	5.63155	8.01E-83
9	SERPINB4	8.07778	7.20E-80
10	ADAMDEC1	3.9596	2.17E-78

Table (9) Top 10 DEGs of non-lesional & normal group of E-GEOD-13355 at p-value <= 0.005 & log2(FC) <= -0.5 | log2(FC) >= 0.5

Sr. no.	SYMBOL	log2(FC)	p-value
1	CNFN	0.55666	9.38E-10
2	C10orf99	0.87757	8.34E-09
3	LYNX1	0.57371	6.93E-08
4	SERTM1	-0.7728	2.63E-07
5	LCE3D	0.79201	3.28E-07
6	NANOS1	-0.6224	3.94E-07
7	ESR1	-0.6143	6.20E-07
8	SLURP1	0.56829	7.95E-07
9	WFDC12	0.69744	2.24E-06
10	SPRR3	0.51881	2.65E-06

Table (10) Top 10 DEGs of E-GEOD-41745 at p-value <= 0.005 & log2(FC) <= -1 | log2(FC) >= 1

Sr. no.	SYMBOL	p-value	log2(FC)
1	SPG21	1.54E-05	3.53706
2	MFAP3L	5.41E-05	-2.842762
3	YBX1	9.12E-05	7.37371
4	PDIA6	0.000125	3.63602
5	CBR1	0.000155	-3.060063
6	NTRK2	0.000157	3.49598
7	ME2	0.00018	1.85502
8	SRSF5	0.000217	4.70286
9	UBE2D3	0.000225	2.26445
10	SFPQ	0.000239	-5.11403

Table (11) Top 10 DEGs of E-GEOD-67785 at p-value <= 0.005 & log2(FC) <= -1 | log2(FC) >= 1

Sr. no.	SYMBOL	p-value	log2(FC)
1	CLEC7A	0	2.86162
2	AKR1B10	4.44E-16	5.15586
3	AKR1B10P1	4.44E-16	2.86703
4	RP11-382A20.1	9.99E-16	2.87976
5	FABP5P2	1.44E-15	3.0129
6	CTD-2262B20.1	1.55E-15	2.96053
7	FABP5P1	1.78E-15	3.05194
8	FABP5P10	1.78E-15	2.94867
9	FABP5P11	2.00E-15	3.04139
10	FABP5P7	2.22E-15	3.06304

Table (12) Differentially expressed miRNAs of E-GEOD-32868 at p-value <= 0.005 & log2(FC) <= -1 | log2(FC) >= 1

Sr. no.	ID	log2(FC)	p-value
1	hsa-miR-106a_st	4.0105	0.00298
2	hsa-miR-132_st	3.5585	0.00175
3	hsa-miR-152_st	3.13315	0.00336
4	hsa-miR-20a_st	4.90297	0.00202
5	hsa-miR-31_st	7.08244	0.00016
6	hsa-miR-671-3p_st	3.22601	0.00433

Table (13) Differentially expressed miRNAs of E-GEOD-34536 at p-value <= 0.005 & log2(FC) <= -1 | log2(FC) >= 1

Sr. no.	ID	log2(FC)	p-value
1	hsa-miR-101	-2.8946	0.00288
2	hsa-miR-140-3p	-1.3379	0.00234
3	hsa-miR-145	-1.8969	0.0003
4	hsa-miR-21*	2.98574	0.00458
5	hsa-miR-214	-2.0344	0.00198
6	hsa-miR-26a	-1.0902	0.00215
7	hsa-miR-30a	-2.1478	8.42E-05
8	hsa-miR-30a*	-3.7529	6.78E-05
9	hsa-miR-31	4.35729	0.00398
10	hsa-miR-378	-1.9361	0.00255
11	hsa-miR-497	-2.0821	0.0005
12	hsa-miR-766	2.43959	0.00228

Table (14) Top 10 DEGs of insitu & normal group of E-GEOD-42677 at p-value <= 0.005 & log2(FC) <= -1 | log2(FC) >= 1

Sr. no.	SYMBOL	log2(FC)	p-value
1	CACNA1C	-1.9489	1.14E-17
2	DTX2P1-UPK3BP1-PMS2P11 (pseudogene)	3.6266	2.32E-16
3	SLC30A3	2.69861	3.18E-16
4	PTH1R	-2.5023	6.99E-16
5	PIP5K1B	-2.1764	1.51E-15
6	UBN1	2.35567	2.57E-15
7	SPDEF	2.74612	4.49E-15
8	METTL7A	-2.2854	6.85E-15
9	SPOCK2	2.22223	2.66E-14
10	FLT4	-1.9591	2.78E-14

Table (15) Top 10 DEGs of invasive & normal group of E-GEOD-42677 at p-value <= 0.005 & log2(FC) <= -1 | log2(FC) >= 1:

**Sr. no.**	**SYMBOL**	**log2(FC)**	**p-value**
1	CACNA1C	-1.9935	7.50E-18
2	DTX2P1-UPK3BP1-PMS2P11 (pseudogene)	3.68792	1.70E-16
3	SLC30A3	2.72379	2.68E-16
4	PIP5K1B	-2.1167	2.51E-15
5	PTH1R	-2.3249	2.70E-15
6	SPDEF	2.69968	6.14E-15
7	METTL7A	-2.28	7.15E-15
8	UBN1	2.22175	7.51E-15
9	SPOCK2	2.29075	1.53E-14
10	FLT4	-1.9104	4.39E-14

Table (16) Top 10 DEGs of invasive & insitu group of E-GEOD-42677 at p-value <= 0.005 & log2(FC) <= -1 | log2(FC) >= 1

**Sr. no.**	**SYMBOL**	**log2(FC)**	**p-value**
1	ATP7A	-1.9055	9.44E-06
2	TGM3	-2.6629	4.89E-05
3	MEIS1	-1.7233	6.98E-05
4	LAMA3	1.59134	0.00024
5	LPAR1	1.52881	0.00029
6	PXN	1.0625	0.00031
7	ZNF174	-1.7149	0.00057
8	S100A3	2.0714	0.00057
9	CD48	1.5485	0.00079
10	NOL7	1.31439	0.00088

Table (17) Top 10 DEGs of E-MTAB-5678 at p-value <= 0.005 & log2(FC) <= -1 | log2(FC) >= 1:

**Sr. no.**	**SYMBOL**	**p-value**	**log2(FC)**
1	RNA5SP88	3.07E-08	-1.94672
2	RN7SL418P	4.11E-08	-1.85651
3	RPL7P7	6.16E-08	-1.81256
4	ZEB2	1.35E-07	-2.12295
5	SCARNA10	1.51E-07	-6.40948
6	PGAP2	1.78E-07	3.525768
7	RP11-887P2.5	1.92E-07	-1.58596
8	FAM3C	2.03E-07	5.961166
9	FNDC3B	2.15E-07	-1.62563
10	VCAN	2.99E-07	-2.37457

Table (18) Top 10 DEGs of GSE84293 at p-value <= 0.005 & log2(FC) <= -1 | log2(FC) >= 1

Sr. no.	SYMBOL	p-value	log2(FC)
1	PPARGC1A	4.41E-07	-2.23056
2	MSRB3	7.30E-07	-1.2894
3	CGNL1	8.23E-07	-2.52465
4	CRYAB	1.59E-06	-2.03383
5	FABP5P11	2.12E-06	1.25539
6	PTHLH	3.39E-06	3.900443
7	LIPH	3.82E-06	-1.71228
8	FZD7	3.89E-06	-1.19911
9	SLC35A3	4.66E-06	-1.66683
10	KRT16	8.00E-06	2.980582
