# Supplementary figures and images for: A Computational Systems Analyses to Identify Biomarkers and Mechanistic Link in Psoriasis and Cutaneous Squamous Cell Carcinoma
Source: Front Immunol. 2021 Jun 18;12:662528. doi: 10.3389/fimmu.2021.662528 (PMC8276676; doi:10.3389/fimmu.2021.662528)

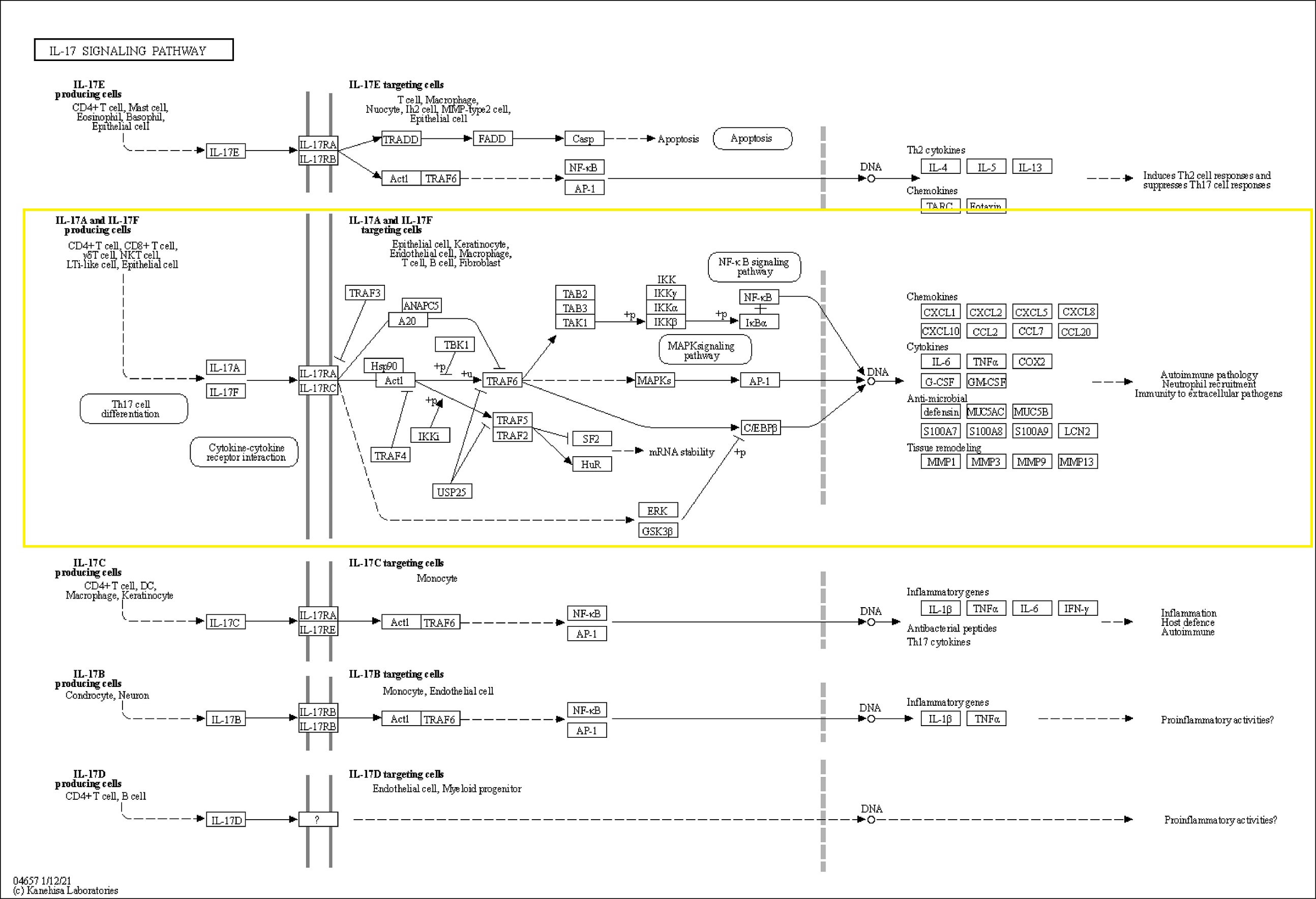

Supplement: Supplementary file 2 [file Image_1.jpeg]
